# Sudemycin E influences alternative splicing and changes chromatin modifications

**DOI:** 10.1093/nar/gku151

**Published:** 2014-03-11

**Authors:** Paolo Convertini, Manli Shen, Philip M. Potter, Gustavo Palacios, Chandraiah Lagisetti, Pierre de la Grange, Craig Horbinski, Yvonne N. Fondufe-Mittendorf, Thomas R. Webb, Stefan Stamm

**Affiliations:** ^1^Department of Molecular and Cellular Biochemistry, University of Kentucky, 741 South Limestone, Lexington, KY 40536, USA, ^2^Department of Chemical Biology and Therapeutics, St. Jude Children's Research Hospital, 262 Danny Thomas Place, Memphis, TN 38105, USA and ^3^GenoSplice Technology, Hôpital Saint-Louis, Av Claude Vellefaux, 75010 Paris, France

## Abstract

Sudemycin E is an analog of the pre-messenger RNA splicing modulator FR901464 and its derivative spliceostatin A. Sudemycin E causes the death of cancer cells through an unknown mechanism. We found that similar to spliceostatin A, sudemycin E binds to the U2 small nuclear ribonucleoprotein (snRNP) component SF3B1. Native chromatin immunoprecipitations showed that U2 snRNPs physically interact with nucleosomes. Sudemycin E induces a dissociation of the U2 snRNPs and decreases their interaction with nucleosomes. To determine the effect on gene expression, we performed genome-wide array analysis. Sudemycin E first causes a rapid change in alternative pre-messenger RNA splicing, which is later followed by changes in overall gene expression and arrest in the G2 phase of the cell cycle. The changes in alternative exon usage correlate with a loss of the H3K36me3 modification in chromatin encoding these exons. We propose that sudemycin E interferes with the ability of U2 snRNP to maintain an H3K36me3 modification in actively transcribed genes. Thus, in addition to the reversible changes in alternative splicing, sudemycin E causes changes in chromatin modifications that result in chromatin condensation, which is a likely contributing factor to cancer cell death.

## INTRODUCTION

Almost all human polymerase II transcripts undergo alternative pre-messenger RNA (pre-mRNA) splicing, which increases the number of proteins that can be encoded in the genome. Exons in pre-mRNA are recognized by the spliceosome, a macromolecular complex composed of five small RNAs and at least 170 proteins (1). An exon is defined by its two splice sites and the branch point, which are only weakly conserved in mammals. The spliceosome assembles around exons in a step-wise manner. First, U1 snRNP binds to the 5′ splice site, followed by binding of splicing factor 1 to the branch point, which increases U2AF binding to the 3′ splice site, stabilizing the entry of U2 snRNP and the release of splicing factor 1. In this spliceosomal A complex, the branch point adenosine is recognized by the U2 snRNP through an interaction between the U2 component SF3B1/(SAP155) and U2AF (2). This stabilization is ATP dependent and allows the inclusion of the U4/U5/U6 snRNPs, leading to the formation of the assembled spliceosome in the B complex. Further rearrangements allow the catalysis of the splicing reaction in the C complex in two transesterification reactions (3). The U2 snRNP undergoes structural changes during the splicing reaction and releases its SF3 complex after the first catalytic splicing step (4).

Alternative exon recognition occurs predominantly cotranscriptional and is therefore mechanistically coupled to other events in gene expression. For example, a fast-moving RNA polymerase promotes alternative exon skipping (5,6). There is accumulating evidence that chromatin structure is linked to exon selection. Nucleosomes are associated with DNA sequences that encode exons (7,8). Histone modification, such as H3K4me3, assists in the recruitment of U2 snRNP components to sites of active transcription, which could promote exon recognition (9). It is possible that pre-mRNA splicing affects chromatin changes, in return, as DNA encoding exons are characterized by the H3K36me3 modification. This modification is lower in DNA corresponding to alternative exons, when compared with constitutive exon. Because on average, alternative exons of the same pre-mRNA assemble fewer spliceosomes than the constitutive exons, this suggests that the activity of the spliceosome is reflected in the H3K36me3 modification.

Reflecting the central role of alternative pre-mRNA splicing in gene expression, abnormal splicing patterns are frequently associated with human diseases. Deregulated splicing patterns are a hallmark of cancer, but the reason for this deregulation is not fully understood (10–12). The tumor microenvironment is generally hypoxic and more acidic than normal tissue, which can result in the pathological generation of protein isoforms supporting metastasis (13). Several protein isoforms generated by alternative splicing are crucial for cancer progression and are the subject of experimental therapeutic intervention. For example, the RON tyrosine kinase gene can generate a constitutively active kinase due to the skipping of an alternative exon (14). The exon, controlled by the splicing factor SF2/ASF, determines the epithelial to mesenchymal transition, which determines the invasiveness of cancer cells (15). Another hallmark of cancer cells are changes in their chromatin structure (16). The recent demonstration that chromatin structure globally influences the localization and availability of splicing factors (17) indicates that missplicing observed in cancer could originate in an altered nuclear structure.

Two naturally occurring compounds, FR901464 and pladienolide B, have been shown to affect pre-mRNA splicing *in vivo* and to suppress tumor growth (18). FR901464 has been derivatized to generate spliceostatin A (19), which targets the U2 snRNP component SF3B, and modulates alternative splicing *in vitro* (19). Further analysis has shown that spliceostatin A inhibits spliceosome assembly in the B complex after a pre-spliceosomal complex has been formed (20). Spliceostatin A influences the interaction of SF3B1 with the pre-mRNA, leading to a nonproductive recruitment of U2 snRNP, which affects a subset of 3′ splice sites (21), explaining why *in vivo* spliceostatin A targets certain alternative splicing events.

Sudemycin E is a refined totally synthetic analog of FR901464 (22). This compound selectively stops the growth of tumors in mice and preferably targets cancer cells, sparing nonneoplastic cells. Similar to spliceostatin A, it changes alternative splicing (23).

Here, we analyzed the effect of sudemycin E on cancer cells. Sudemycin works via a two-stage mechanism, first reversibly affecting alternative splicing of ∼7.5% of alternative exons due to a dissociation of U2 snRNPs. Possibly due to the dissociation, sudemycin E decreases the binding of U2 snRNPs to nucleosomes. As a likely result, the H3K36me3 chromatin mark in DNA corresponding to altered alternative exons is decreased. Several hours later, we found general changes in gene expression, not just in alternative splicing. Together, these events lead to chromatin condensation and an arrest in the G2 stage of the cell cycle.

## MATERIALS AND METHODS

### Cell culture

Rh18 cells, established at St. Jude Children’s Research Hospital (24), were grown in RPMI 1640 medium (Roswell Park Memorial Institute) supplemented with 10% (v/v) heat-inactivated fetal calf serum at 37°C in 5% CO_2_. HEK293 cells (Sigma) were grown in high glucose Dulbecco’s modified Eagle’s medium containing 10% (v/v) heat-inactivated fetal calf serum, at 37°C in 5% CO_2_. Human skin primary fibroblasts (GM 00498D, Coriell Institute) were grown in minimum essential medium Eagle containing 10% (v/v) heat-inactivated fetal calf serum, at 37°C in 5% CO_2_.

### Cell viability

Viability was tested by metabolizing 3-[4,5-dimethylthiazol-2-yl]-2,5-diphenyltetrazolium bromide (MTT) by mitochondrial dehydrogenases (Sigma): 1 000 000 cells were treated with the compounds at the times indicated and were subsequently analyzed with MTT according to the manufacturer’s protocol. The MTT staining obtained from cells receiving just dimethyl sulfoxide (DMSO) control were set as 100% signal.

### Biotinylation pull down

Streptavidin beads (Life Technologies) were washed three times with phosphate-buffered saline (PBS) buffer and then precleared with 10% bovine serum albumin for 30 min at 4°C. The precleared beads were incubated with 25 µl of DMSO or 10 mM biotinylated sudemycin E and HeLa nuclear (DundeeCellProducts) extract in 2× immunoprecipitation buffer [IP, 10 mM Tris–HCl (pH 7.4), 150 mM NaCl, 10 mM KCl] overnight. The next day, beads were separated from unbound fractions, washed two times and mixed with 90 µl of 1× sodium dodecyl sulphate (SDS) sample (50 mM Tris–HCl, pH 6.8, 2% SDS, 10% glycerol, 1% β-mercaptoethanol, 12.5 mM ethylenediaminetetraacetic acid and 0.02% bromophenol blue) buffer. Sixty microliters of unbound and washed fractions were mixed with 30 µl of 3× SDS sample buffer. All fractions were boiled for 5 min and loaded onto 10% SDS–polyacrylamide gels.

### Nucleosome preparation

HEK293 cells were pelleted at 1000 rpm at 4°C and washed in ice-cold PBS buffer. The cell pellet was resuspended in ice-cold NP-40 lysis buffer [10 mM Tris (pH 7.4), 10 mM NaCl, 3 mM MgCl_2_, 0.5% NP-40, 0.15 mM spermine and 0.5 mM spermidine] and incubated on ice for 5 min. The solution was centrifuged at 3000 rpm for 10 min. The nuclear fraction (pellet) was then resuspended in 1 ml of ice-cold micrococcal nuclease digestion buffer [10 mM Tris (pH 7.4), 15 mM NaCl, 60 mM KCl, 0.15 mM spermine, 0.5 mM spermidine and 1 mM CaCl_2_] and digested with 50 U/ml micrococcal nuclease at 37°C for 12 min. The reaction was stopped by addition of EDTA and NP-40 to an end concentration of 0.01 M and 0.1%, respectively.

### Sudemycin E handling

Sudemycin E was dissolved in DMSO at 10 mM final concentration. Rh18, HEK293 and fibroblasts were plated and cultured for 24 h to 80% confluence before sudemycin E treatment. The cells were treated with 1 or 10 µM sudemycins (up to 48 h) or vehicle (DMSO) equal to solvent.

### Determination of sudemycin E concentration

After sudemycin E addition to the medium, the medium and cells were collected and extracted with acetonitrile. The extracted solution was analyzed by ultra-performance liquid mass spectrometry coupled to mass spectrometry [UPLC-MS/MS (TQ detector)]. The parent ion of sudemycin E was quantitatively determined by comparing it with a standard curve.

### Flow cytometry

In all, 1 × 10^6^ HEK293 cells were fixed in 70% ethanol, incubated for 30 min at 4°C, treated with RNase A (250 µg/ml) and stained with propidium iodide 10 µg/ml (Sigma) for 30 min at 37°C. Cell cycle analysis was performed on a flow cytometer (FACSCalibur, BD Biosciences).

### Microscopy

HEK293 cells were seeded into 4-well chambers (Thermo Scientific). After 24 h, cells were treated with 0, 4, 6 and 24 h with sudemycin E and then washed with PBS. Four percent of paraformaldehyde in PBS, pH 7.4, was used for 10 min to fix cells. Then, the cells were incubated for 10 min with PBS containing 0.25% Triton X-100 (PBST). One percent bovine serum albumin in PBST was used to block unspecific antibody binding. Cells were incubated for 1 h in SF3B1 antibody, washed three times with PBS and then incubated for another hour with fluorescein isothiocyanate (FITC) anti-rabbit antibody. Finally, cells were stained with 4',6-diamidino-2-phenylindole (DAPI) and analyzed by confocal microscopy (Nikon A1R-A1 Confocal Microscope System).

### Glycerol gradient analysis

Nuclear extract from HeLa cells was loaded onto a linear 4-ml 15–30% glycerol gradient prepared in G150 buffer (20 mM HEPES, pH 7.9, 150 mM NaCl, 1.5 mM MgCl_2_, 0.5 mM dithioerythritol). After centrifugation in a Beckman SW60Ti rotor at 35 000 rpm (corresponding to 165 000*g*), at 4°C for 15 h, the gradient was fractionated into 26 fractions of 150 µl each. 30 µl of 3× SDS sample buffer were added to 60 µl of each fraction. The samples were boiled for 5 min, and 15 µl of each fraction was loaded onto 10% SDS–polyacrylamide gels. Anti SF3B1 antisera was obtained from Abcam. To estimate the Svedberg (S) values, we used conalbumin (molecular weight 75 000 D; 5.4 S), aldolase (15 800 D, 11.5 S) and ferritin (440 000, 17 S), all from GE healthcare.

### Nucleosome immunoprecipitation

Nucleosomes were immunoprecipitated in 2× IP buffer [10 mM Tris–HCl (pH 7.4), 150 mM NaCl, 10 mM KCl] for 14–16 h at 4°C using specific antibodies to H3 (AbCam) or no antibody (No Ab). The following day, beads were separated from unbound fractions and washed several times. The unbound (Input), H3 and No Ab fractions were collected, mixed with 3× SDS sample buffer, boiled for 5 min and loaded onto 10% SDS–polyacrylamide gels.

### Chromatin immunoprecipitations

Rh18 and HEK293 cells were lysed and sheared by sonication in 0.1% NP-40 in PBS (Sigma) lysis buffer to generate cellular chromatin fragments of 400–500 bp. The chromatin was immunoprecipitated for 14–16 h at 4°C using specific antibodies to H3K36me3 and H3K27me3 (AbCam). After the incubation, chromatin immunoprecipitates were purified and then 2 µl of each sample was analyzed by real-time polymerase chain reaction (PCR).

### Real-time PCR

The real-time PCR was carried out in the Strategene Mx3005P (Agilent Technologies), using SYBR green reagent (Life Technologies). The relative expression was estimated as follows: 2^Ct(reference)^^ − ^^Ct(sample)^, where C_t_ (reference) and C_t_ (sample) were input DNA and specific histone modification chromatin, respectively. For each experiment, at least three immunoprecipitations were analyzed.

### Reverse transcriptase-polymerase chain reaction

Total RNA from Rh18, HEK293 and fibroblast cells was extracted using GenElute Mammalian Total RNA Miniprep kit (Sigma) according to the manufacturer’s instructions. cDNAs were synthesized from 1 µg of each RNA using SuperScript® III Reverse Transcriptase (Life Technologies). Reverse transcriptase-polymerase chain reaction (RT-PCR) experiments were carried out using 1 µl of each cDNA as template and specific primers. Products were visualized on gel electrophoresis after ethidium bromide staining. Primers used were as follows:

RPp30 _FOR:GAGGCCTGGCTTTTGAACTT; RPp30 _REV:CCTTGGCGTCACTTTCAGAG; DUSP11_FOR:GACATCAAGTGCCTGATGATGA; DUSP11_REV:ATGTCCCCGGCACCTATT; SRRM1_FOR:GACTCTGGCTCCTCCTCCTC; SRRM1_REV:GGACTTCTCCTCCGTCTACCA; PAPOLG_FOR:AAGAGATCCCATTCCCCATC; PAPOLG_REV:TGCGTGATGTATCAATAGTTGGA; MLH3_FOR:TTATTGCCTGTTTGATGAGCAC; MLH3_REV:TCCTTTGTTCCTCTGTCACTGTT; ß-ACTIN_FOR:AGAGCTACGAGCTGCCTGAC; ß-ACTIN_REV:GGATGTCCACGTCACACTTC; ADAT1_FOR:ATGGCCAGGTGGTCTTCATA; ADAT1_REV:GTCACTTGCACCGGCTTATC; DNAJB7_FOR:CAGCAACAGAGATCCCCCTA; DNAJB7_REV:AGCACCAACTGTCACCACAA; PRPF39_FOR:ACCCTGGTGATCCTGAGACA; PRPF39_REV:GAAGCTAATTCCCTTCGCAAC; cRPP30 _FOR:TATATCTAGTGCTGCAGAAAGG; cRPP30 _REV:GCCTAAAGAAAGTGGGGATAA; cDUSP11_FOR:TTGTTTTGTTATTTAGGTTGGA; cDUSP11_REV:ACTCACTCCTATAATACCAACACTT; cSRRM1_FOR:ATCGCCAGTGACTAAAAG; cSRRM1_REV:AATCTAAGTTCAAATAAGGGTC; cPAPOLG_FOR:CTGCCTACATAGGCCTATCGA; cPAPOLG_REV:GCGAGAGTCGTCTCTTAGAT; cMLH3_FOR:CTGGGATTCAAACATATGGGATA; cMLH3_REV:TCCAGACGTATACGCTCAT; cADAT1_FOR:TATTTTGGGAGGTTGAGG; cADAT1_REV:ATCAAAAAATTTTTTAAAATAAAATCT; cDNAJB7_FOR:GTGTAATGTTTATTATTTTGTTTGAGA; cDNAJB7_REV:ATTCAAACGATTCTCCTATCTC; cPRPF39_FOR:GATTTTTGGGAGGGTTAGG; cPRPF39_REV:CCTAACCGAAAATAACACTTCA; AURKB_sFOR:ATGACCGGAGGAGGATCTAC; AURKB_sREV:GATGGACCTCCAGCTACAAG; AURKB_FOR:ACATCTTAACGCGGCACTTC; AURKB_REV:TTGTCTTCCTCCTCAGGGAGG.

### Array analysis

RNA was isolated using Qiagen kits. Its quality was determined by RNA integrity number analysis, and samples with an RNA integrity number > 9.5 were used following the Affymetrix labeling procedure.

For the analysis, the signal from Affymetrix human junction arrays (HJAY) was normalized using the ‘Probe scaling’ method. The background was corrected with ProbeEffect from GeneBase (25). The gene expression index was computed from probes that were selected using ProbeSelect from GeneBase (25). The gene expression signals were computed using these probes. Genes were considered expressed if the mean intensity was ≥500. Genes were considered regulated if (i) they were expressed in at least one condition (i.e. VPA and/or control); (ii) the fold-change was ≥1.5, which is above the noise level seen in cells (26); and (iii) the unpaired *t*-test *P*-value between gene intensities was ≤0.05. For each probe, a splicing index was computed. Unpaired *t*-tests were performed to determine the difference in probe expression between the two samples as described previously (27). Probe *P*-values in each probeset were then summarized using Fisher’s method. Using annotation files, splicing patterns (cassette exons, 5′/3′ alternative splice sites and mutually exclusive exons) were tested for a difference between isoforms, selecting the ones with a minimum number of regulated probeset (with a *P* ≤ 0.01) in each competing isoform (at least one-third of ‘exclusion’ probesets have to be significant; at least one-third of ‘inclusion’ probesets have to be significant and show an opposite regulation for the splicing index compared with the ‘exclusion’ probesets). For example, for a single cassette exon, the exclusion junction and at least one of the three inclusion probesets (one exon probeset and two inclusion junction probesets) have to be significant and have to show an opposite regulation for the splicing index. The exon junction arrays contained 13 150 alternative cassette exons, 6517 alternative 5′/3′ exons and 1145 mutually exclusive exons (20 812 alternative exons). A total of 33 395 genes are taken into account.

## RESULTS

### Sudemycin E binds to SF3B1 and is toxic for some cancer cells

**Figure 1.**
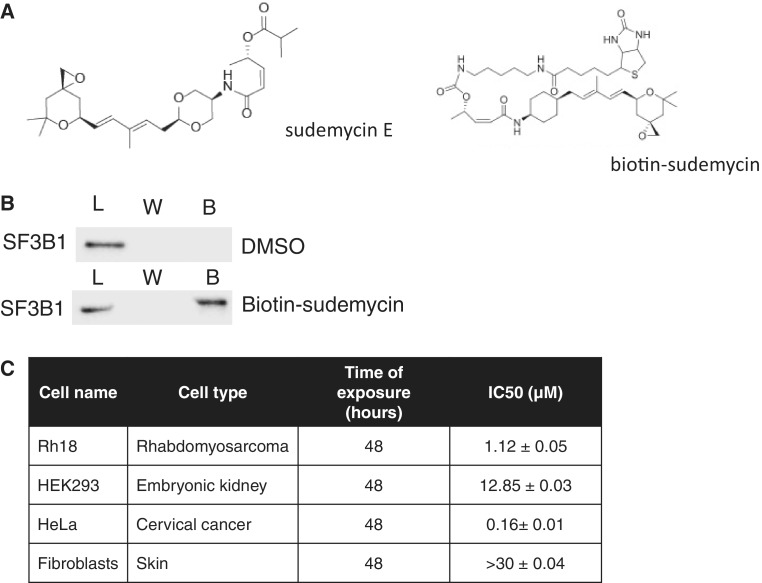
Sudemycin E binds to SF3B1 and is toxic for cancer cell lines. (**A**) Structure of sudemycin E (left) and its biotinylated derivative (right). (**B**) Binding of biotinylated sudemycin to SF3B1. Biotinylated sudemycin was bound to streptavidin coupled to magnetic beads, and incubated with HeLa nuclear extract, followed by washing with PBS. The bound SF3B was eluted by boiling with 1% SDS, 10 mM Tris and SF3B1 detected by western blot. L: load, W: wash, B: bound sudemycin. Load corresponds to the total input. Ten percent of the input was loaded. (**C**) IC50 determined for Rh18, HEK293, HeLa and primary fibroblast cells using the MTT assay; *n* = 4. Sude E: sudemycin E.

Sudemycin E (Figure 1A, left) is chemically related to spliceostatin A, a methylated derivative of FR901464, which is a natural product that binds to the U2 component SF3B1 and modulates splicing (19). Sudemycin E is a more stable chemically refined totally synthetic analog of FR901464 and its derivative spliceostatin A, which is much more chemically stable than these compounds (22). Sudemycin E also has much less stereochemical complexity than FR901464 or spliceostatin A, as it contains only three stereocenters compared with nine in those compounds (22). This means that sudemycin E and analogs are more amenable to medicinal chemistry structure-activity studies and scale-up to large quantities that will ultimately be needed for possible future clinical studies. Sudemycin E selectively stops the growth of tumors in mice and preferably targets cancer cells, sparing nonneoplastic cells. Similar to spliceostatin A, it changes alternative splicing (23).

We first tested whether sudemycin E also binds to SF3B1 and used a biotinylated derivative of sudemycin in pull-down experiments. After binding biotinylated sudemycin (Figure 1A, right) to streptavidin-coated magnetic beads, the beads were incubated with HeLa nuclear extract, washed and protein was eluted. Using western blot, we detected binding of SF3B1 to sudemycin E-coated beads. This demonstrates sudemycin binding to SF3B1, as expected from its chemical structure.

Sudemycin E causes the death of certain cancer cells, while being generally nontoxic in nontransformed cells (23). To understand the selectivity for cancer cells, we determined the IC50 of sudemycin E toxicity in four cell lines: Rh18, a rhabdomyosarcoma cell line representing the most common soft tissue sarcoma in children; HEK293 cells, adenovirus-transformed human embryonic kidney cells; HeLa cells, a cervical cancer cell line; and human primary skin fibroblasts. After sudemycin E treatment, death of cells becomes noticeable at 24 h and is more pronounced at 48 h, where we determined the IC50 using the MTT assay. As shown in Figure 1C, the IC50 ranges from 0.16 to >30 µM. HeLa cells are most sensitive to sudemycin E, with an IC50 ∼160 nM after 48 h of treatment, followed by Rh18 cells with an IC50 of 1.12 µM. In contrast, HEK293 cells and primary fibroblasts are 10–30 times less sensitive to sudemycin E, reflecting the selectivity of the drug for cancer cells.

### A short incubation with sudemycin E is sufficient to cause cell death

The IC50 in Rh18 cells depends on the time the drug is present in the cells and ranges from >30, 5 and 0.8 µM after 2, 8 and 72 h of treatment, respectively (23). Therefore, we asked whether cells have to be continuously exposed to sudemycin E for a cytotoxic effect to occur or whether the drug triggers a cellular response that causes the death of cells.

**Figure 2.**
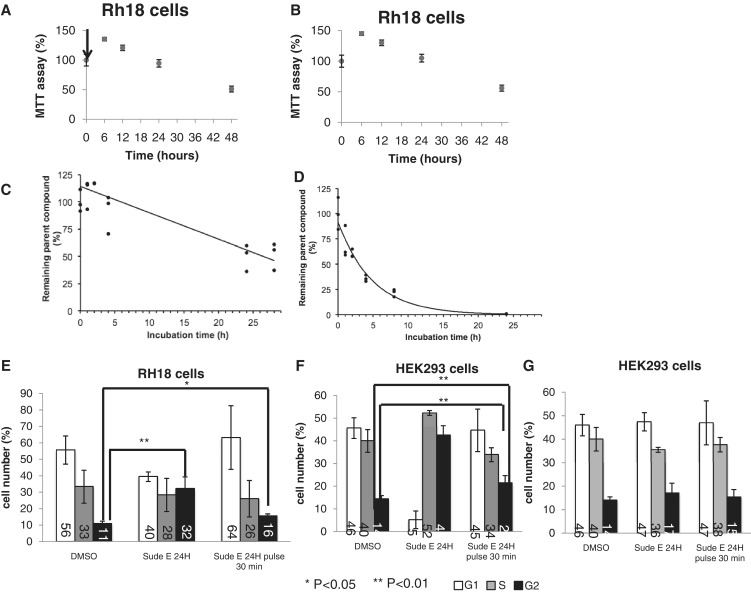
A short treatment with sudemycin E is sufficient to cause cell death. (**A**) Survival of Rh18 cells in the presence of 1 µM sudemycin; sudemycin was added at time 0 and cell viability measured by the MTT assay, n = 4; arrow indicates time of wash-out. (**B**) Survival of Rh18 cells after 30-min incubation with 1 µM sudemycin, followed by a wash out of the drug through a change in medium. The cell viability was measured by the MTT assay, *n* = 4. (**C**) Stability of sudemycin in cell culture medium. One micromolar sudemycin was incubated in cell culture medium, and the amount of sudemycin E was determined using mass spectrometry after acetonitrile extraction. (**D**) Stability of sudemycin in the presence of cells. Sudemycin was incubated with Rh18 cells, and the amount of sudemycin was measured after acetonitrile extraction. (**E**) Cell cycle phases of Rh18 cells after 24 h DMSO treatment, 24 h sudemycin E treatment (1 µM) and after a 30-min sudemycin E pulse, followed by medium change and 23.5 h incubation. (**F**) Cell cycle phases of HKE293 cells after 24 h DMSO treatment, 24 h sudemycin E treatment (10 µM) and after a 30-min sudemycin E pulse, followed by medium change and 23.5 h incubation. (**G**) Similar to B, but 1 µM sudemycin was used. One micromolar sudemycin does not cause considerable cell death in HEK293 cells. *P < 0.05; **P < 0.01; *n* > 4.

Rh18 cells were treated with 1 µM sudemycin, which was washed out by changing the cell culture medium after 30 min. This was followed by 48 h culture without the drug (Figure 2A). The survival of these cells was compared with cells treated with 1 µM sudemycin for 48 h, where we left sudemycin E on the cells (Figure 2B). We did not observe significant differences in cell viability, measured by the MTT assay between the two conditions. Using mass spectrometry, we determined sudemycin E concentration in medium and found that it rapidly disappears (Figure 2C and D).

The natural compound FR901463 that provided the scaffold for sudemycin E was shown to arrest M-8 cells in the G2/M phase of the cell cycle (18). Therefore, we asked whether we observe similar effects after short sudemycin E incubation times. We performed cell cycle analysis after cells were treated for 24 h with sudemycin E or treated for only 30 min, followed by a medium change that removes sudemycin and a subsequent incubation for 23.5 h. Sudemycin E was used at a concentration corresponding to the IC50, 1 µM for Rh18 cells and 10 µM for HEK293 cells (Figure 2E and F). In addition, a 1 µM concentration that shows low toxicity for HEK293 cells was used and had no significant effect on the cell cycle (Figure 2G).

As shown in Figure 2E and F, when used at concentrations that are toxic for cells, we observed an arrest of both Rh18 and HEK293 cells in G2. When used for 24 h, the amount of cells in G2 increased ∼3-fold. When used for only 30 min, we found an increase of ∼50% in both cell lines, which was statistically significant (Figure 2E and F). In contrast, using 1 µM sudemycin, a concentration that is not cytotoxic for HEK293 cells did not cause significant changes (Figure 2G). The data indicate that sudemycin E causes an arrest of cells in the G2 phase of the cell cycle, which can also be seen after a 30-min incubation time.

Together, the data show that sudemycin has cytotoxic effects after a short incubation time, and that it triggers events that cause later cell death, as opposed to continuous blocking a cell activity that is needed for survival.

### Sudemycin E breaks up the U2 complex *in vitro*

**Figure 3.**
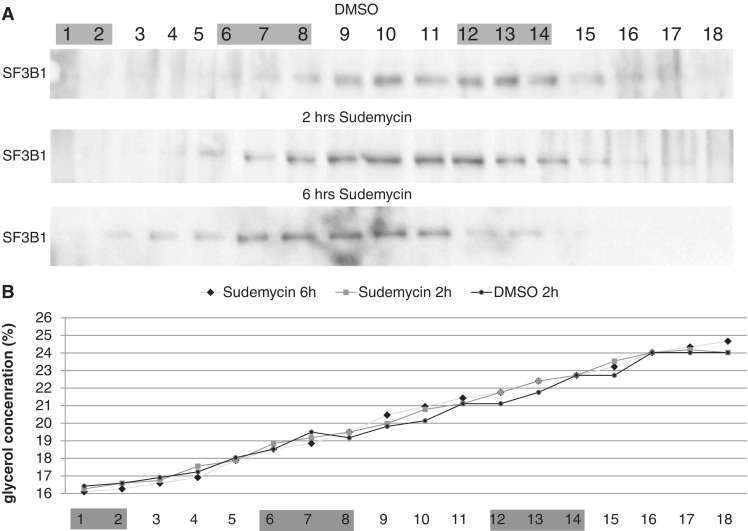
Sudemycin causes a dissociation of the U2 complex. (**A**) Hela nuclear extract was incubated with DMSO or 10 µM sudemycin for 2 and 6 h and loaded on 15–30% glycerol gradients. The gradient (total volume: 4 ml) was divided into 20 fractions and analyzed by western blot using and antiserum against SF3B1. The gray-shaded areas indicate the sedimentation of marker proteins, fractions 1–2: conalbumin 5.4 S; 6–8: aldolase 11.5 S; and 12–14: ferritin 17 S. (**B**) The glycerol concentration was measured by refractometry in all gradients. Each line corresponds to a gradient used in Figure 3A.

SF3B1 is part of the U2 complex, a macromolecular complex of at least 11 proteins assembling on the small U2 RNA (SM proteins, SF3B130, SF3B155, SF3a60, SF3B145, SF3a120, SF3a66, U2-A’, U2-B’, SFb49, SF3B14b and SF3B10). The U2 complex is largely remodeled during the splicing reaction, with the loss of SF3A and SF3B proteins during splicing catalysis (28). Therefore, we determine the influence of sudemycin E on the U2 complex using HeLa nuclear extract. HeLa nuclear extract was incubated with 10 µM sudemycin and separated on glycerol gradients, as previously described (29). As shown in Figure 3A, after treatment, we detect SF3B1 no longer in the U2 complex of about 17 S, but in lighter fractions, corresponding to 11–12 S. U2 snRNPs extracted under high salt conditions sediment at 12 S, as several proteins present under splicing conditions are lost from the U2 snRNP (29).

This suggests that sudemycin E does not simply bind to SF3B1 found in the U2 complex, but causes its dissociation. Other than in the loaded sample, there was no sudemycin E present in the gradient, suggesting that the effect of sudemycin on the U2 complex is irreversible.

### SF3B1 physically interacts with histone H3

It is well established that splicing and transcription are coupled (30). Deep sequencing experiments indicate an association of U2 snRNPs with histone H3 (31). Therefore, we tested whether histone H3 can physically interact with components of the U2 complex.

**Figure 4.**
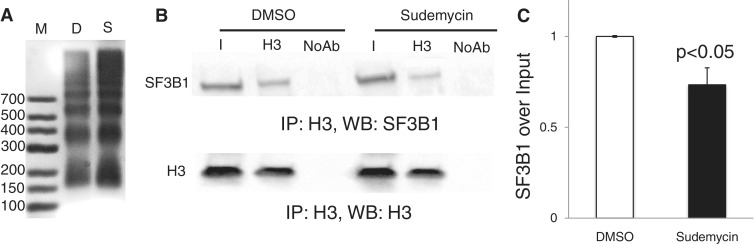
Sudemycin E influences the binding of SF3B1 to purified nucleosomes. HEK293. Cells were treated with 10 µM sudemycin for 6 h, and nucleosomes were prepared. (**A**) Visualization of the nucleosomal preparation using an ethidium bromide-stained agarose gel after protein extraction. (**B**) Detection of SF3B1 after nucleosome precipitation. Nucleosomes were immunoprecipitated using an anti H3 antiserum, separated on PAGE and SF3B1 was detected using western blot. (**C**) Quantification of four experiments.

Because chromatin is largely insoluble, we purified oligo-nucleosomes from sudemycin-treated and naïve Hela cells by performing limited chromatin nuclease digestion (Figure 4A). Mono- and dinucleosomes were immunoprecipitated with an anti H3 antiserum, and bound SF3B1 was detected using western blot. As shown in Figure 4B and C, we observe coprecipitation between the U2 complex SF3B1 and nucleosomes, demonstrating a physical interaction. Importantly, in the presence of sudemycin E, this interaction is reduced by 25% (*P *< 0.05, *n* = 4).

These data indicate a physical interaction between nucleosomes and SF3B1. It is likely that SF3B1 is in a complex with U2 that is bound by nucleosomes. The reduction of SF3B1 binding to nucleosomes could be caused by a dissociation of the U2 complex that we observed in gradient centrifugation.

### A 6-h treatment with sudemycin E predominantly changes alternative splicing

**Figure 5.**
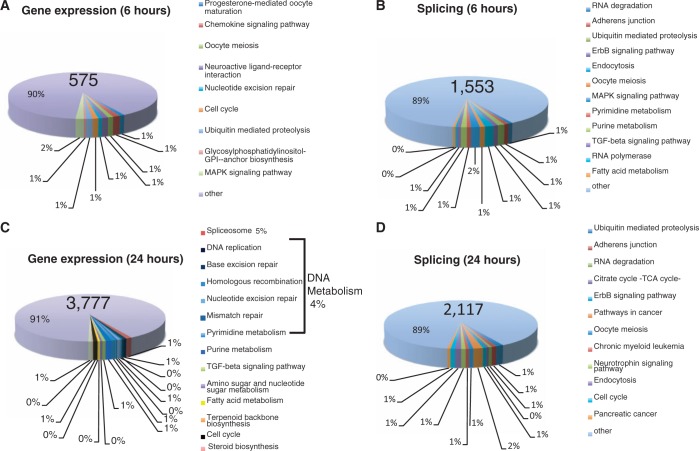
Array analysis to determine expression changes caused by sudemycin E. Changes in gene expression are shown. The pie chart shows the strongest deregulated Kyoto Encyclopedia of Genes and Genomes pathways. (**A**) Rh18 cells were treated with 1 µM sudemycin E for 6 h. Overall, 575 genes changed their overall expression. (**B**) The array data showed that 1553 alternative exons were changed (1314 cassette exons, 121 mutually exclusive exons and 118 alternative 5′ and 3′ splice sites). The array data are in Supplementary Figure S2. (**C**) Rh18 cells were treated with 10 µM sudemycin E for 24 h. In all, 3777 genes showed changes in overall gene expression and (**D**) 2117 alternative splicing events were changed (1761 cassette exons, 143 mutually exclusive exons, 213 alternative 5′/3′ splice sites). The array data are in Supplementary data as Excel files.

To determine the molecular effects of sudemycin treatment, we performed genome-wide exon junction array analysis. First, we treated Rh18 cells with 1 µM of sudemycin E for 6 h. The concentration of 1 µM was chosen because it is in the IC50 range for Rh18 cells, which is 1.12 ± 0.05 µM (Figure 1C). We choose 6 h, as there is no measurable cell death at this time point, which allows detecting early consequences of sudemycin E action. The array analysis indicated that sudemycin E changes 1553 alternative exons (Figure 5B), which is expected for a substance inhibiting the U2 snRNP. Unexpectedly, we observed changes in general gene expression for 575 genes (Figure 5A). Gene ontology (GO) pathways that were affected the strongest were nitrogen metabolic processes, nitrogen compound processes and nucleoside synthesis. Because there is no observable cell death at 6 h of treatment (Figure 2A and B), it is likely that the changes observed here represent the first cellular response to sudemycin, which affects alternative exons in most biological pathways. These data are in agreement with findings for spliceostatin (21) that rapidly changes splice site selection of selected alternative exons.

### A 24-h treatment with sudemycin E changes predominantly gene expression

To determine the changes in gene expression under conditions when Rh18 cells start dying due to sudemycin E treatment, we added 10 µM of the drug for 24 h. Because sudemycin E rapidly disappears in the presence of Rh18 cells (Figure 2C and D), we choose a higher sudemycin E concentration to ensure that sudemycin E is present throughout the treatment time. Unexpectedly, we observed more changes in overall gene expression (3777; Figure 5C) than in alternative splicing (2117; Figure 5D). The most affected Kyoto Encyclopedia of Genes and Genomes pathway was the spliceosome, where 43 of 128 genes were affected. Forty-one of these genes were upregulated. About 4% of all changes affected genes acting in DNA metabolism, indicating a deregulation in DNA repair and replication. Most of these genes (2108) were upregulated in expression; 1669 were downregulated.

The data sets obtained after 6 and 10 h of sudemycin E treatment are mostly not overlapping. The most striking difference is a change in expression of 43 of 128 genes acting in the spliceosome, none of which was changed after 6 h of treatment.

Together, the data suggest that sudemycin E acts in two stages: first, it causes changes in alternative splicing. Later, the drug changes mainly gene expression, which affects the spliceosome and DNA metabolism the strongest.

### Early changes in alternative splicing occur gradually and are reversible

We next validated changes observed in the arrays by RT-PCR, using primers in constitutive exons flanking an alternative cassette exon. The overall validation rate was >85% (7 of 8), when splicing events with a high confidence of prediction were used (*P* < 10^−^^10^).

**Figure 6.**
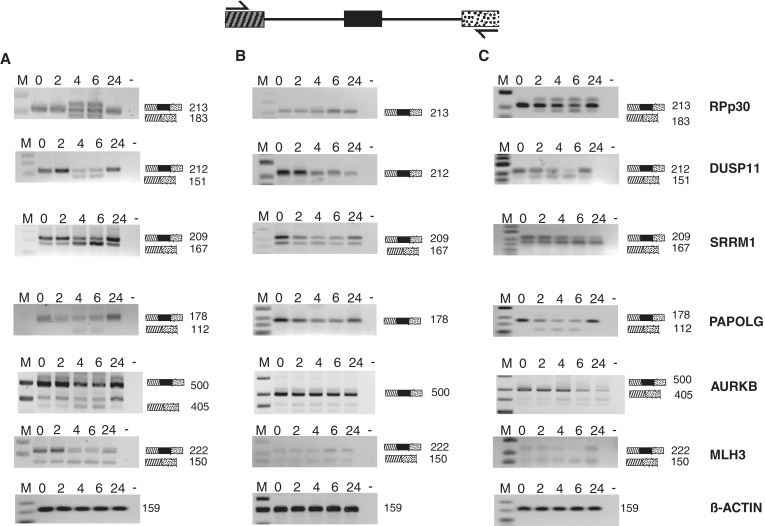
Effect of sudemycin E on alternatively spliced genes in Rh18 and HEK293 cells. (**A**) Rh18 cells were treated with 1 µM of sudemycin E for 2, 4, 6, 24 h, and alternative splicing of transcripts for RPp30, DUSP11, SRRM1, PAPOLG and MLH3 was assessed by PCR. The PCR products are schematically indicated, numbers indicate their sizes. M: marker; 0, no drug; -, water. Beta-actin was used as a loading control. (**B**) HEK293 cells were exposed to 1 µM sudemycin E. (**C**) HEK293 cells were exposed to 10 µM sudemycin E.

Because sudemycin E appears to act in a step-wise manner, we determined changes in alternative splicing in a time course by RT-PCR. We first analyzed Rh18 cells using 1 µM sudemycin, a concentration around the IC50 value. Sudemycin E was added at time 0, and cells were tested after 2–24 h, without changing the medium. For the genes tested, changes in alternative splicing increased gradually for 6 h and reverted to the original splicing pattern after 24 h (Figure 6A), consistent with the decrease of sudemycin E (Figure 2D).

We next used the same 1 µM sudemycin E concentration for HEK293 cells and did not observe significant changes in alternative splicing (Figure 6B). However, when using the higher concentration of 10 µM, which is comparable with the IC50 of sudemycin E in HEK293 cells, we observed changes in alternative splicing (Figure 6C), similar to the ones observed in Rh18 cells. In all concentrations and cell used, the alternative splicing patterns revert to the original condition after 24 h. This could be due to the loss of sudemycin, as we found that >90% of sudemycin E is degraded after 12 h in cell culture (Figure 2D).

The data indicate that sudemycin E rapidly changes splicing, as expected for a compound interfering with the U2 component SF3B1. These changes do not persist in cells, most likely because sudemycin E decays and cells repair the damaged U2 particles.

### Thirty minutes of sudemycin E treatment cause changes in alternative splicing

**Figure 7.**
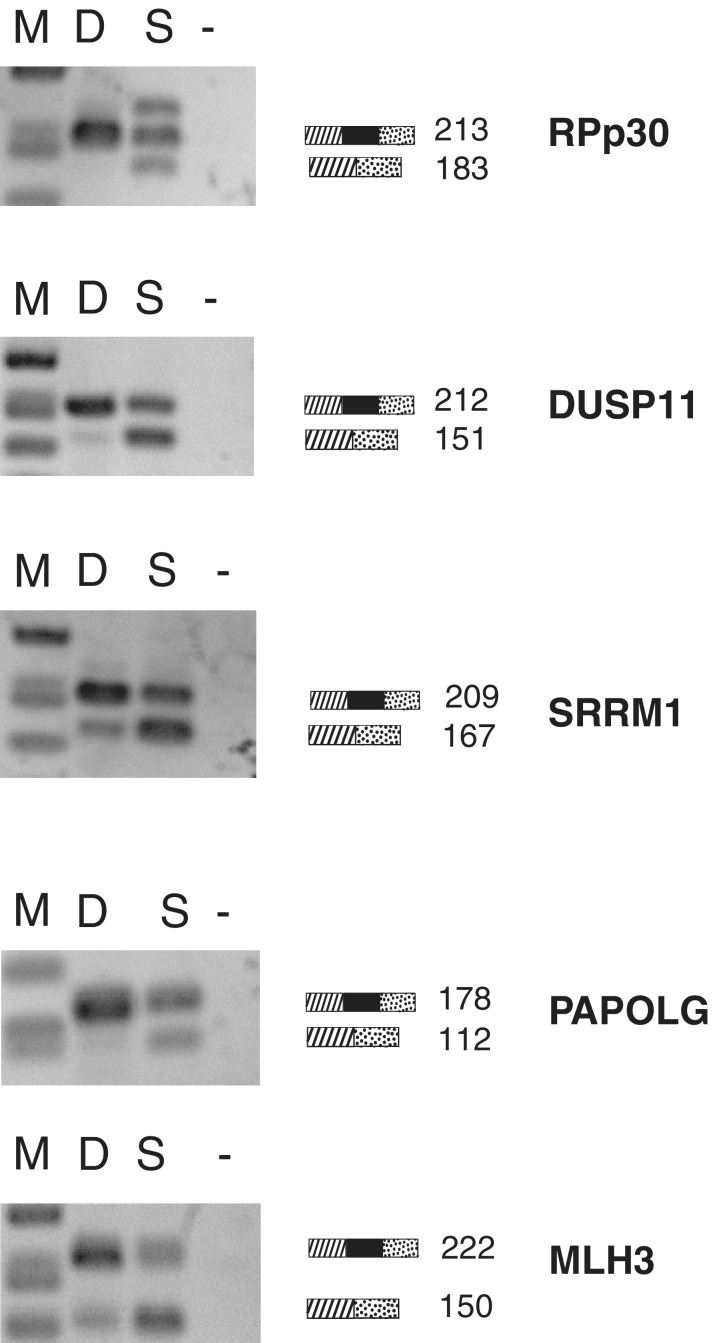
Changes in alternative splicing after a short incubation with sudemycin E. Rh18 cells were incubated with 1 µM sudemycin E for 30 min. The drug was removed by medium change and cellular RNA was analyzed after 6 h by RT-PCR. D: DMSO control, S: sudemycin E.

Because sudemycin E caused death of cells 48 h after a short incubation time (Figure 2A), we investigated whether 30 min of sudemycin E treatment causes changes in alternative splicing. Rh18 cells were treated with 1 µM sudemycin E for 30 min. The drug was then removed by a change in medium. Changes in splicing were determined after 6 h by RT-PCR. As shown in Figure 7, this short pulse is sufficient to trigger a change in alternative splicing. This indicates that sudemycin causes rapid changes in cells, leading to detectable changes in alternative splicing later. Changes in alternative splicing are most likely detected after enough of the mRNA present before drug treatment decayed, which results in their detection after a few hours.

### Changes in gene expression caused by sudemycin E persist after drug treatment

**Figure 8.**
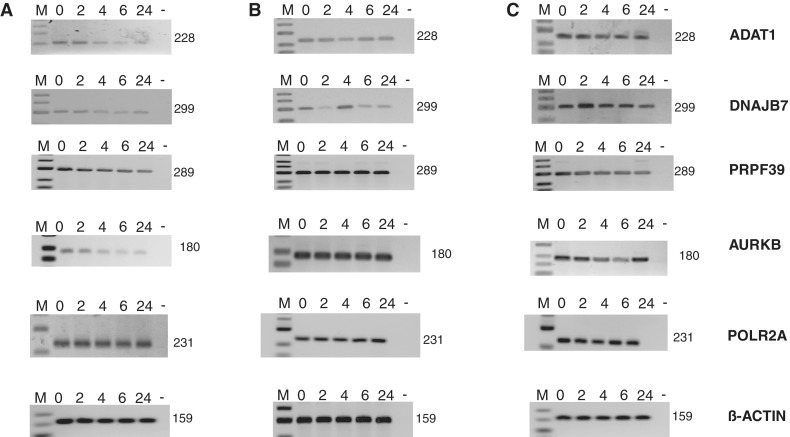
Effect of sudemycin E on overall gene expression. (**A**) Rh18 cells were treated with 1 µM of sudemycin E for 2, 4, 6, 24 h, and alternative splicing of transcripts for RPp30, DUSP11, SRRM1, PAPOLG and MLH3 was assessed by PCR. Numbers are the size of the PCR products. M, marker; 0, no drug; -, water. (**B**) HEK293 cells were exposed to 1 µM sudemycin E. (C) HEK293 cells were exposed to 10 µM sudemycin E.

An unexpected finding of sudemycin E treatment was the deregulation of general gene expression (validation rate by RT-PCR 60%, 8 of 13). We analyzed several of these changes in detailed time courses using RT-PCR. As shown in Figure 8A, in Rh18 cells treated with 1 µM sudemycin, gene expression changes gradually over time, similar to changes in alternative splicing. However, most of the changes in expression do not revert to the original splicing pattern when sudemycin E is degraded after 24 h (Figure 2C). Similar to changes in splicing, most analyzed genes in HEK293 cells are not changed using 1 µM sudemycin E (Figure 8B). However, HEK293 cells change splicing patterns in response to sudemycin E at a concentration of 10 µM, near their particular IC50, similar to Rh18 cells (Figure 8C and B). These data suggest that changes in gene expression evoked by sudemycin E are generally not as rapidly reversible as changes in alternative splicing.

### Sudemycin E changes histone modifications

The persistent change in gene expression after sudemycin E treatment suggested a change in chromatin modification. It has been shown that U2 snRNPs associate with H3K36me3 modifications (31) and we could detect binding of the U2 component SF3B1 to nucleosomes, supporting the idea that U2 directly interacts with chromatin. Furthermore, sudemycin E decreases SF3B1 binding (Figure 4C). This indicates that sudemycin E treatment changes the U2/nucleosome interaction, possibly because the drug causes a dissociation of the U2 complex (Figure 3).

**Figure 9.**
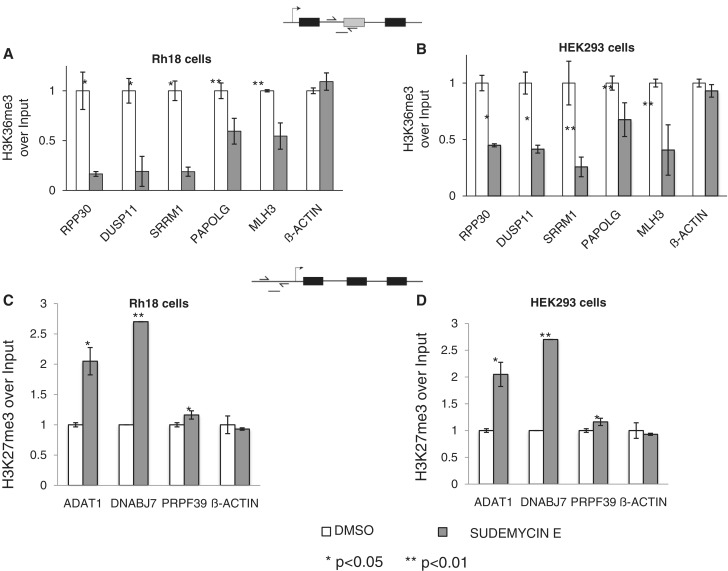
Change of chromatin modifications after sudemycin treatment. (**A**) Rh18 cells were treated with 1 µM sudemycin E for 6 h, and chromatin was immunoprecipitated with anti H3K36me3. The amplicons used are schematically shown on top. (**B**) Similar immunoprecipitations in HEK293 cells treated with 10 µM sudemycin E. (**C**) Rh18 cells were treated with 1 µM sudemycin E for 6 h and chromatin was immunoprecipitated with anti H3K27me3. The amplicons in the annotated promoter regions used for real-time PCR are schematically shown on top. (**D**) A similar experiment was performed in HEK293 cells, using 10 µM sudemycin E for 6 h.

We tested an influence of sudemycin E on histone modifications using chromatin immunoprecipitations. Cells were treated with sudemycin E for 6 h, and chromatin was immunoprecipitated using an H3K36me3 antibody. We then measured the DNA corresponding to pre-mRNA intron/exon borders that are influenced by alternative splicing using real-time PCR. As shown in Figure 9A and B, we found a marked decrease of H3K36me3 in both Rh18 and HEK293 cells when regions within the gene were analyzed. Similar to other experiments, we used 1 µM sudemycin E for Rh18 cells and 10 µM for HEK293 cells. Beta-actin as a nonaffected gene showed no effect.

Because we detected changes in the overall gene expression after sudemycin E treatment, we analyzed histone modifications in promoter regions. We focused on the H3K27me3 modifications, which generally represses gene expression (32). As shown in Figure 9C and D, after 6 h of sudemycin E treatment, there is an increase in the H3K27me3 modification. This correlates with the decrease in the overall gene expression that we observed using RT-PCR. However, the changes in the promoter regions are not as uniformly strong as changes in the H3K36me3 modifications seen at exon junctions. Again, the nonregulated gene beta actin showed no change in modification.

Collectively, the data indicate that sudemycin E causes a change in chromatin modifications. The drug decreases modifications that favor an open chromatin conformation.

### Sudemycin E causes chromatin condensation

**Figure 10.**
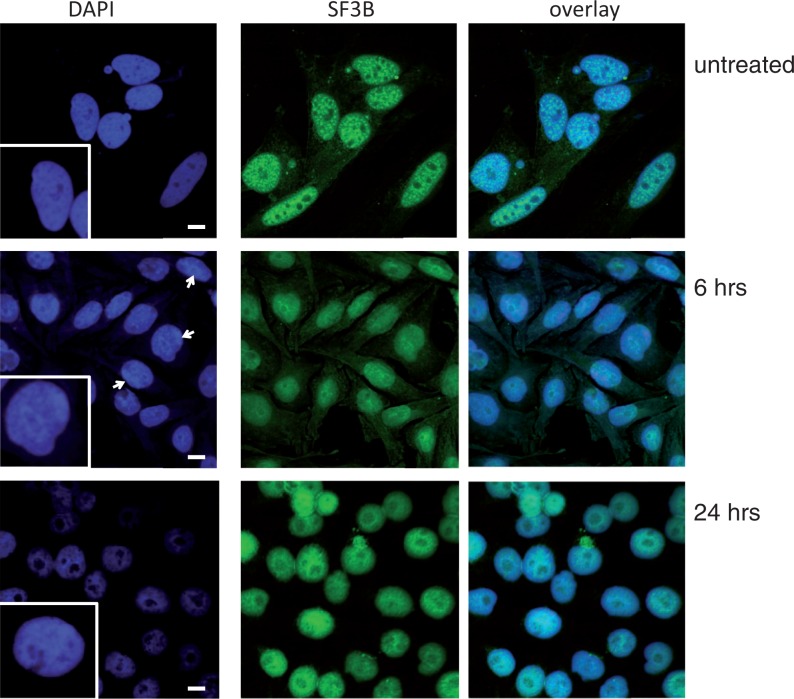
Change of global chromatin after sudemycin treatment. HEK293 cells were treated with 10 µM sudemycin for 6 or 24 h and analyzed by confocal microscopy. After 6 h, ∼30% of the cells showed chromatin condensations, indicated by focal DAPI staining (arrows). The cell nuclei were stained with DAPI and with an antiserum against SF3B1.

H3K36 trimethylation increases transcription and generally opens chromatin. Because we observed a strong decrease of H3K36me3 modification in target genes after sudemycin E treatment, we asked whether sudemycin E causes chromatin condensation. We stained Rh18 cells after 6 and 24 h of sudemycin E treatment. Similar to spliceostatin A, sudemycin E disrupts SF3B1-containing speckles. In addition, we found that treated cells showed an increase of DAPI staining in large foci (Figure 10), suggestive of chromatin condensation.

## DISCUSSION

Splicing modulators related to FR901464 show promise as anti-cancer drugs. However, the reason for their selectivity is not understood. Here we investigate the mechanism of sudemycin E, a simple chemical derivative of FR901464 and its related compound spliceostatin A. Sudemycin analogs are less complex (containing only three stereocenters rather than the nine in FR901464 and spliceostatin A), which facilitates its synthesis and structure–activity relationship analysis. Importantly, sudemycin E is more potent in killing cancer cells and tumors than in its toxicity toward normal human cells (22). FR901464 is chemically unstable in aqueous solution, with a half-life of 45 min in cell culture medium (19). This raises the question whether the change in alternative splicing observed for spliceostatin A (21) is the sole or the major cause for the death of cancer cells.

**Figure 11.**
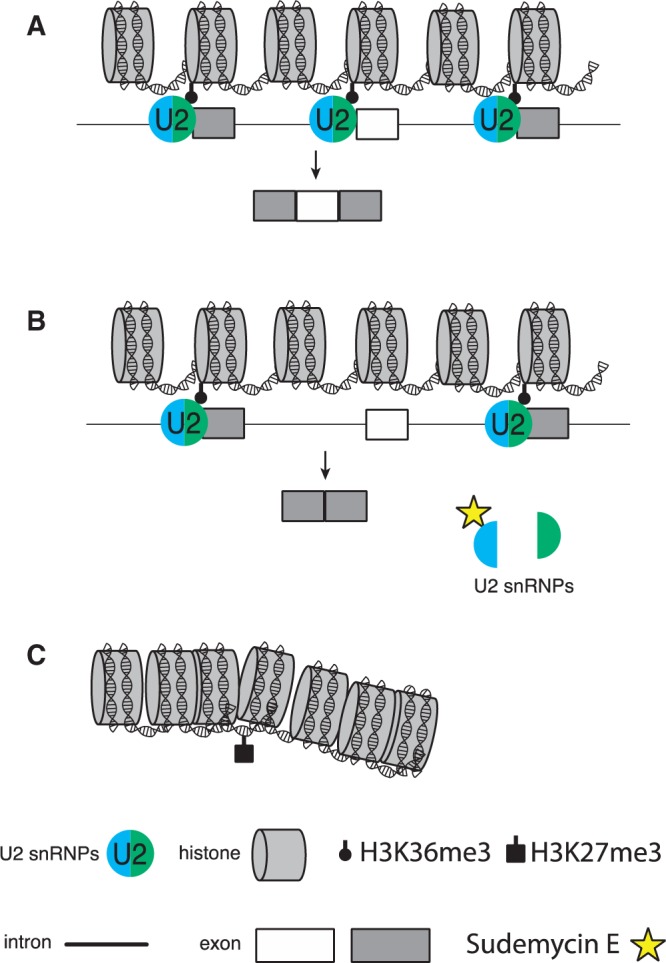
Model for sudemycin E treatment. (**A**) In actively transcribed genes, U2 snRNP interacts with histones, which stabilizes the H3K36me3 modification. U2 snRNP can interact with histones that are close to alternative exons. (**B**) Sudemycin E binds to the U2 component SF3B1 and causes a dissociation of the complex. Sudemyin affects alternative exons that are likely dependent on base pairing with U2 snRNA (white exon). The H3K36me3 modification is removed near these exons. (**C**) The loss of the H3K36me3 modification causes chromatin condensation, and possibly spreads through chromatin, leading to the observed changes in gene expression.

Our results suggest that ongoing pre-mRNA splicing is necessary to keep the epigenetic state of cancer cells. Sudemycin causes structural changes of U2 snRNPs, which likely result in epigenetic changes. We propose that these epigenetic changes strongly contribute to the selectivity of sudemycin for cancer cells (Figure 11).

### Sudemycin E changes alternative splicing

To determine molecular targets of sudemycin E, we performed genome wide array analysis. We found that after 6 h of treatment, 1553 alternative exons are changed, which represent 7.46% of the alternative exon on the array. In agreement with studies using reporter genes (23), this clearly shows that sudemycin E does not globally block pre-mRNA splicing, but predominantly modulates a subset of alternative splicing events. This modulation of alternative splicing, but not a global inhibition in cell culture, has also been reported for spliceostatin A (21). We did not detect statistical significant changes in sequence or exon length in the exons affected by sudemycin E, but it is likely that similar to spliceostatin A (21), alternative exons that show suboptimal base pairing to U2 snRNA are affected.

Using identified splicing events, we compared different cell lines in their response to sudemycin E. Surprisingly, we found that the splicing patterns are influenced in a similar way independently of sudemycin’s toxicity for the cell. The only difference is the concentration of sudemycin E needed to change splicing, which needs to be higher in sudemycin-insensitive cells to alter splice site selection. This suggests that sudemycin can enter both sensitive and insensitive cells.

We followed the effect of sudemycin E on alternative splicing in time course experiments and observed for most genes that the strongest changes occur between 2 and 4 h. Unexpectedly, the splicing patterns revert to the original mode after 24 h, when we can measure an arrest in cell cycle. We observed the loss of most of sudemycin E after 24 h of incubation in cells, which could be due to a cellular degradation system or chemical reactivity of sudemycin E. Therefore, it is likely that sudemycin E reversibly inhibits the spliceosome in cells, which will function normally after the drug has been removed.

Unexpectedly, a 30-min incubation of sudemycin, followed by drug removal, is enough to obtain a change in alternative splicing after 6 h and an arrest in the cell cycle after 24 h. The 6-h time delay could reflect the time it takes to degrade mRNAs present at the time of sudemycin E treatment, as only after these RNAs are removed, changes in alternative pre-mRNA splicing will be visible. The exact molecular mechanism and the kinetics of sudemycin E action on U2 snRNP remain to be determined. It is possible that sudemycin E inhibits the biological activity of SF3B1 before it leads to the dissociation of the U2 complex. The U2 snRNP undergoes rearrangements during the splicing reaction, and it is thus possible that the binding site for sudemycin E changes during the reaction. Because only a subset of alternative exons is affected by sudemycin E, it is likely that sudemycin E affects only U2 snRNPs that are actively engaged in the splicing reaction of these exons. U2 complexes participating in splicing of other exons could be more stable due to other proteins present in exon enhancer complexes.

Finally, when sudemycin is removed after a 30-min pulse, the alternative splicing patterns revert to the original splicing mode. Because the cells still die, it is likely that factors other than the splicing changes contribute to cell death.

### Sudemycin E causes a dissociation of the U2 complex *in vitro* and influences U2 nucleosome interaction

FR901464 binds to the spliceosomal component SF3B1 (19). SF3B1 is part of the U2 complex, formed by the association of at least 18 proteins with the U2 snRNA. The SF3 complex composed of SF3B and SF3A dissociates before the first step of splicing, after the spliceosome has been properly assembled on the pre-mRNA (4).

We found that a biotinylated sudemycin derivative binds to SF3B1, which was expected due to its pharmacophore similarity to spliceostatin A. Because the U2 snRNP dissociates during the splicing reaction, we tested the influence of sudemycin E on the U2 snRNP complex *in vitro,* and found that after sudemycin treatment, SF3B1 immunoreactivity is present in lighter gradient fractions, indicating a dissociation of the U2 complex. When spliceosomes are prepared under higher salt conditions, several proteins are stripped off the mature U2 snRNP, resulting in a particle sedimenting around 12 S (29). After 6 h of sudemycin treatment, we detect SF3B1 in fractions corresponding to 11–12 S. It remains to be determined whether sudemycin E has the same effect on U2 snRNPs as high salt. Isolated SF3B1 protein is expected to sediment around 11 S assuming a globular structure, but it is not clear what structure the protein has when sudemycin E is bound.

The composition of the U2 snRNP complexes formed after sudemycin E treatment remains to be determined, but it is possible that the drug recapitulates the natural dissociation of the SF3 complex from the U2 snRNP that occurs during the splicing reaction.

The majority, an estimated 80%, of pre-mRNA splicing occurs co-transcriptionally in human cells (33). It is well established that chromatin modifications influence constitutive and alternative pre-mRNA splicing (5). For example, exons correspond to specific chromatin marks (8,34) and histone modifications influence alternative splicing by recruiting auxiliary factors to the nascent pre-mRNA (35). In return, RNA guides enzymes to chromatin modifying complexes (36). To test an interaction between SF3B1 and chromatin, we prepared soluble mono-, di- and tri-nucleosomes under native conditions and determined SF3B1 binding. We detected SF3B1 after immunoprecipitations of these preparations, suggesting an interaction between the U2 snRNPs and nucleosomes. Because the preparations contain DNA and RNA, it is possible that they are nucleic acid mediated. Importantly, sudemycin-treated cells show significantly less binding of SF3B1 to nucleosomes. Together with our finding that sudemycin E causes a dissociation of U2 snRNP, these data indicate that the intact U2 snRNP preferentially binds to nucleosomes.

A functional influence of sudemycin E on U2 snRNP–nucleosome interaction was apparent in chromatin immunoprecipitations using K36me3-modified histone H3. It has been earlier shown that the presence of SF3B3 influences this mark, which is increased at exon/intron junctions (31,37). In all genes tested, we found a decrease in the H3K36me3 modification after cells were treated with sudemycin E at a concentration that causes cell cycle arrest. H3K36me3 is associated with open chromatin in the bodies of active genes. We postulate that U2 snRNPs participate in maintaining the H3K36me3 modification, possibly by direct interaction with nucleosomes in active genes (Figure 11A). The loss of U2 snRNP activity caused by sudemycin leads rapidly to a change in alternative splicing (Figure 11B), which is reversed after the drug is either removed or degraded. Sudemycin E causes a dissociation of U2 snRNP, which likely influences chromatin modifications, such as H3K36me3, which decreases. Because H3K36me3 is generally associated with open chromatin, its loss could lead to a condensation of chromatin (Figure 11C). We postulate that this chromatin condensation spreads in the nucleus affecting expression of multiple genes that we detected in the array experiments after 24 h.

Similar to stem cells, cancer cells have generally more dynamic chromatin, in part characterized by abundant H3K36me3 marks (38,39). A condensation of chromatin, caused in part by the loss of H3K36me3 marks in active genes, will reprogram cancer cells to die. In this model, chromatin changes strongly contribute to the selectivity of sudemycin for cancer cells.

There are now several diseases known to be caused by aberrant expression of splicing factors, for example, spinal muscular atrophy (loss of SMN), amyotrophic lateral sclerosis (mutants of FUS) and retinitis pigmentosa (loss of constitutive splicing factors PRP8 and PRP18 (40). It is not understood why a change in pre-mRNA splicing causes cell death in these diseases and most investigations focused on a change in splicing isoforms. Our data suggest the possibility that changes in pre-mRNA processing could lead to chromatin changes that ultimately cause cell death.

## SUPPLEMENTARY DATA

Supplementary Data are available Online.

## FUNDING

Funding for open access charge: NIH [CA140474, GM083187 and 5P20RR020171-08]; Cancer Center Core [CA21765]; American Lebanese Syrian Associated Charities (ALSAC) and St. Jude Children’s Research Hospital (SJCRH).

*Conflict of interest statement*. None declared.

## Supplementary Material

Supplementary Data
