# Supplementary material for: Sudemycin E influences alternative splicing and changes chromatin modifications
Source: Nucleic Acids Res. 2014 Mar 11;42(8):4947–61. doi: 10.1093/nar/gku151 (PMC4005683; doi:10.1093/nar/gku151)
Supplement: Supplementary Data [file supp_42_8_4947__index.html]

Sudemycin E influences alternative splicing and changes chromatin modifications — Sudemycin E influences alternative splicing and changes chromatin modifications — Supplementary Data 

# Sudemycin E influences alternative splicing and changes chromatin modifications

## Supplementary Data

files

**Files in this Data Supplement:**

- Supplementary Data - zip file
