# Supplementary material for: Sudemycin E influences alternative splicing and changes chromatin modifications
Source: Nucleic Acids Res. 2014 Mar 11;42(8):4947–61. doi: 10.1093/nar/gku151 (PMC4005683; doi:10.1093/nar/gku151)
Supplement: Supplementary Data [file supp_gku151_nar-03224-y-2013-File012.zip › NAR-03224-2013 Suppl files/Supplemental_Data.docx]

# Supplemental Data

## **Supplemental Figure 1:**

Change of gene expression and alternative splicing after 6 hours of Sudemycin E treatment.

## **Supplemental Figure 2:**

Change of gene expression and alternative splicing after 24 hrs of Sudemycin E treatment.
