# Supplementary material for: Sudemycin E influences alternative splicing and changes chromatin modifications
Source: Nucleic Acids Res. 2014 Mar 11;42(8):4947–61. doi: 10.1093/nar/gku151 (PMC4005683; doi:10.1093/nar/gku151)
Supplement: Supplementary Data [file supp_gku151_nar-03224-y-2013-File012.zip › NAR-03224-2013 Suppl files/Supplemental_Data_2.pdf]

Customer name:	Stefan STAMM	
Project name:	10µm vs. Neg	
Organism:	Human	
Chip:	Affymetrix Hjay (Junction Array)	
Analysis level:	Alternative 5'/3' splice site	
Date:	2010-10-05	
GS contact (name/email/phone):	Pierre DE LA GRANGE	pierre.delagrange@genosplice.com

List of the 213 differentially regulated Alternative 5'/

Rank	Confident	Transcript Cluster
1	HIGH	TC0300778
2	HIGH	TC0701679
3	HIGH	TC0800135
4	HIGH	TC1700465
5	HIGH	TC1701420
6	HIGH	TC1600420
7	HIGH	TC1200059
8	HIGH	TC0100054
9	HIGH	TC1101036
10	HIGH	TC1101036
11	HIGH	TC1101036
12	HIGH	TC1101036
13	HIGH	TC1000744
14	HIGH	TC0401269
15	HIGH	TC0800278
16	HIGH	TC1500220
17	HIGH	TC0400753
18	HIGH	TC1900055
19	HIGH	TC1700397
20	HIGH	TC0501453
21	HIGH	TC0300211
22	HIGH	TC0301452
23	HIGH	TC0900642
24	HIGH	TC2100261
25	HIGH	TC2000806
26	HIGH	TC0901406
27	HIGH	TC0301245
28	HIGH	TC0300997
29	HIGH	TC0100484
30	HIGH	TC1501010
31	HIGH	TC2200546
32	HIGH	TC1900699
33	HIGH	TC1200827
34	HIGH	TC1700988
35	HIGH	TC1700988
36	HIGH	TC1700708
37	HIGH	TC1101036
38	HIGH	TC0900470
39	HIGH	TC0103461

40	**HIGH**	TC0103461
41	**HIGH**	TC1101520
42	**HIGH**	TC0202116
43	**HIGH**	TC0202319
44	**HIGH**	TC0601645
45	**HIGH**	TC1901638
46	**HIGH**	TC1101974
47	**HIGH**	TC0700956
48	**HIGH**	TC0700956
49	**HIGH**	TC0700956
50	**HIGH**	TC1600558
51	**HIGH**	TC0600245
52	**HIGH**	TC0701431
53	**HIGH**	TC2000043
54	**HIGH**	TC1600434
55	**HIGH**	TC0400830
56	**HIGH**	TC1100546
57	**HIGH**	TC0500621
58	**HIGH**	TC0500621
59	**HIGH**	TC0400507
60	**HIGH**	TC1200338
61	**HIGH**	TC1101488
62	**HIGH**	TC0102808
63	**HIGH**	TC2000429
64	**HIGH**	TC1001219
65	**HIGH**	TC0103368
66	**HIGH**	TC1901060
67	**HIGH**	TC0103414
68	**HIGH**	TC1901209
69	**HIGH**	TC1901324
70	**HIGH**	TC1000532
71	**HIGH**	TC1201618
72	**HIGH**	TC1201148
73	**HIGH**	TC0301373
74	**HIGH**	TC0701630
75	**HIGH**	TC0301553
76	**HIGH**	TC0100169
77	**HIGH**	TC1101913
78	**HIGH**	TC1901096
79	**HIGH**	TC1901146
80	**HIGH**	TC0500559
81	**MEDIUM**	TC1000628
82	**MEDIUM**	TC1800422
83	**MEDIUM**	TC1101826
84	**MEDIUM**	TC0100789
85	**MEDIUM**	TC1100134
86	**MEDIUM**	TC1100134
87	**MEDIUM**	TC0301134
88	**MEDIUM**	TC0301134
89	**MEDIUM**	TC1500940
90	**MEDIUM**	TC2000413
91	**MEDIUM**	TC0501558
92	**MEDIUM**	TC0901307
93	**MEDIUM**	TC0600841
94	**MEDIUM**	TC0301067
95	**MEDIUM**	TC1700457

96	MEDIUM	TC1700754
97	MEDIUM	TC0202010
98	MEDIUM	TC1401068
99	MEDIUM	TC0301259
100	MEDIUM	TC1901282
101	MEDIUM	TC1400691
102	MEDIUM	TC1101363
103	MEDIUM	TC0300596
104	MEDIUM	TC0102717
105	MEDIUM	TC0102717
106	MEDIUM	TC1400349
107	MEDIUM	TC0701655
108	MEDIUM	TC0701655
109	MEDIUM	TC1200657
110	MEDIUM	TC2100405
111	MEDIUM	TC0102517
112	MEDIUM	TC0100936
113	MEDIUM	TC1201649
114	MEDIUM	TC1700177
115	MEDIUM	TC2200589
116	MEDIUM	TC1101637
117	MEDIUM	TC1200005
118	MEDIUM	TC0201343
119	MEDIUM	TC0101106
120	MEDIUM	TC0700041
121	MEDIUM	TC1201190
122	MEDIUM	TC0100267
123	MEDIUM	TC1400236
124	MEDIUM	TC1901719
125	MEDIUM	TC1901719
126	MEDIUM	TC1101215
127	MEDIUM	TC1900571
128	MEDIUM	TC1701078
129	MEDIUM	TC1001125
130	MEDIUM	TC1500372
131	MEDIUM	TC2000531
132	MEDIUM	TC0100561
133	MEDIUM	TC0101283
134	MEDIUM	TC0500303
135	MEDIUM	TC0300510
136	MEDIUM	TC0300818
137	MEDIUM	TC0700722
138	MEDIUM	TC0200848
139	MEDIUM	TC0600506
140	MEDIUM	TC1000381
141	MEDIUM	TC0400796
142	MEDIUM	TC0601440
143	MEDIUM	TC1500465
144	MEDIUM	TC1400739
145	MEDIUM	TC0100621
146	MEDIUM	TC1901156
147	MEDIUM	TC1300298
148	MEDIUM	TC1300203
149	MEDIUM	TC0701034
150	MEDIUM	TC1501189
151	MEDIUM	TC1600563

152	MEDIUM	TC0300026
153	MEDIUM	TC1400426
154	MEDIUM	TC0800549
155	MEDIUM	TC1700670
156	MEDIUM	TC1701007
157	MEDIUM	TC1400424
158	MEDIUM	TC0101633
159	MEDIUM	TC0202448
160	MEDIUM	TC0202448
161	MEDIUM	TC0600410
162	MEDIUM	TC0500813
163	MEDIUM	TC0800254
164	MEDIUM	TC1600778
165	MEDIUM	TC0202316
166	MEDIUM	TC0300755
167	MEDIUM	TC1101491
168	MEDIUM	TC0103014
169	MEDIUM	TC0700388
170	MEDIUM	TC0700891
171	MEDIUM	TC0201780
172	MEDIUM	TC0100496
173	MEDIUM	TC0601397
174	MEDIUM	TC0102385
175	MEDIUM	TC0700137
176	MEDIUM	TC2200213
177	MEDIUM	TC0102859
178	MEDIUM	TC1400238
179	MEDIUM	TC1500253
180	MEDIUM	TC1500253
181	MEDIUM	TC0102840
182	MEDIUM	TC1200634
183	LOW	TC1901060
184	LOW	TC2000881
185	LOW	TC1900291
186	LOW	TC0500126
187	LOW	TC1700986
188	LOW	TC1300123
189	LOW	TC0500530
190	LOW	TC0301171
191	LOW	TC1900262
192	LOW	TC2000695
193	LOW	TC1901542
194	LOW	TC1900699
195	LOW	TC0201653
196	LOW	TC0201520
197	LOW	TC1600718
198	LOW	TC0102865
199	LOW	TC2200289
200	LOW	TC0900166
201	LOW	TC0401078
202	LOW	TC0401078
203	LOW	TC0401078
204	LOW	TC0401078
205	LOW	TC0500742
206	LOW	TC0701472
207	VERY LOW	TC1700275

208	**VERY LOW**	TC0800135
209	**VERY LOW**	TC1401158
210	**VERY LOW**	TC2200571
211	**VERY LOW**	TC1400636
212	**VERY LOW**	TC1100313
213	**VERY LOW**	TC0801169

+ 33 (0) 1 57 27 68 39

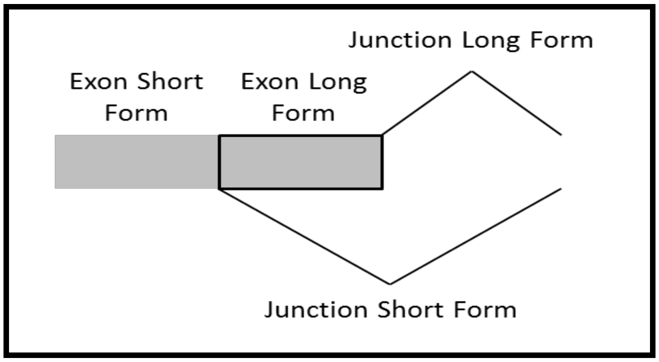

/3' Splice sites

Gene Symbol	Gene Description	Gene ID	UCSC Link	Event Graphic View	Comment
Dtrp1,FLJ32803,HTP	Translocation protein 1	ENSG000000	UCSC	Graphic View	NA
HIPK2	Homeodomain-interact	ENSG000000	UCSC	Graphic View	NA
BMP1,FLJ44432,PCC	Bone morphogenetic p	ENSG000001	UCSC	Graphic View	NA
MAD7,MLX,MXD7,TC	MAX-like protein X (ML	ENSG000001	UCSC	Graphic View	NA
ATXN7L3	ATXN7L3 protein (Frag	ENSG000000	UCSC	Graphic View	NA
---	PAPD5 protein (Fragm	ENSG000001	UCSC	Graphic View	NA
PTMS	Parathymosin|Parathyr	ENSG000001	UCSC	Graphic View	NA
RER1	RER1 protein|RER1 re	ENSG000001	UCSC	Graphic View	NA
MGC18200,MGC4569	Ribonuclease inhibitor	ENSG000000	UCSC	Graphic View	NA
MGC18200,MGC4569	Ribonuclease inhibitor	ENSG000000	UCSC	Graphic View	NA
MGC18200,MGC4569	Ribonuclease inhibitor	ENSG000000	UCSC	Graphic View	NA
MGC18200,MGC4569	Ribonuclease inhibitor	ENSG000000	UCSC	Graphic View	NA
FLJ16452,GDI2,RAB	GDP dissociation inhibi	ENSG000000	UCSC	Graphic View	NA
ELF2,EU32,NERF,NE	E74-like factor 2 (ets d	ENSG000001	UCSC	Graphic View	NA
KIAA0146	---	ENSG000001	UCSC	Graphic View	NA
THBS,THBS1,TSP,TS	Thrombospondin 1 (TH	ENSG000001	UCSC	Graphic View	NA
GAK	Cyclin G-associated kir	ENSG000001	UCSC	Graphic View	NA
AZI,MGC138338,OAZ	Ornithine decarboxylas	ENSG000001	UCSC	Graphic View	NA
ADA2,FLJ12705,KL04	Ional adapter 2-like (AD	ENSG000001	UCSC	Graphic View	NA
CLINT1	Clathrin interactor 1 (Cl	ENSG000001	UCSC	Graphic View	NA
C3orf23	Chromosome 3 open re	ENSG000001	UCSC	Graphic View	NA
C3orf17	Chromosome 3 open re	ENSG000001	UCSC	Graphic View	NA
EXOSC2,RRP4,Rrp4	Exosome complex exo	ENSG000001	UCSC	Graphic View	NA
C21orf6	Chromosome 21 open	ENSG000001	UCSC	Graphic View	NA
MGC31836,PRKCBP	Protein kinase C bindin	ENSG000001	UCSC	Graphic View	NA
FLJ20922,RALGDS,R	Ral guanine nucleotide	ENSG000001	UCSC	Graphic View	NA
LIP4,MCG10,PCBP4	Poly(rC) binding protei	ENSG000000	UCSC	Graphic View	NA
FLJ34707,MGC29784	RNA pseudouridylate s	ENSG000001	UCSC	Graphic View	NA
C1orf164,FLJ10597,R	Chromosome 1 open re	ENSG000001	UCSC	Graphic View	NA
NARG2	NMDA receptor regulat	ENSG000001	UCSC	Graphic View	NA
HPS4,KIAA1667,LE,b	Hermansky-Pudlak syn	ENSG000001	UCSC	Graphic View	NA
BAX	Apoptosis regulator BA	ENSG000000	UCSC	Graphic View	NA
ARA24,Gsp1,RAN,TC	GTP-binding nuclear p	ENSG000001	UCSC	Graphic View	NA
C17orf61,PLSCR3	Chromosome 17 open	ENSG000001	UCSC	Graphic View	NA
C17orf61,PLSCR3	Chromosome 17 open	ENSG000001	UCSC	Graphic View	NA
BPTF,FAC1,FALZ,NU	Fetal Alzheimer antiger	ENSG000001	UCSC	Graphic View	NA
MGC18200,MGC4569	Ribonuclease inhibitor	ENSG000000	UCSC	Graphic View	NA
ZNF462	Zinc finger protein 462|	ENSG000001	UCSC	Graphic View	NA
FLJ20531,ZNF692,Zf	Zinc finger protein 692|	ENSG000001	UCSC	Graphic View	NA

FLJ20531,ZNF692,Zf	Zinc finger protein 692|	ENSG000001	UCSC	Graphic View	NA
D11S636,SF1,ZFM1,Z	Splicing factor 1 (SF1),	ENSG000001	UCSC	Graphic View	NA
ARHGAP2,CHN,CHN	Chimerin (chimaerin) 1	ENSG000001	UCSC	Graphic View	NA
ZNF142	Zinc finger protein 142	ENSG000001	UCSC	Graphic View	NA
REPS1	RALBP1 associated Ep	ENSG000001	UCSC	Graphic View	NA
DKFZp547H236,MEIS	Meis1, myeloid ecotrop	ENSG000001	UCSC	Graphic View	NA
BTBD15	BTB (POZ) domain cor	ENSG000001	UCSC	Graphic View	NA
DKFZp686J14194,FJ	FtsJ homolog 2 (E. coli	ENSG000001	UCSC	Graphic View	NA
DKFZp686J14194,FJ	FtsJ homolog 2 (E. coli	ENSG000001	UCSC	Graphic View	NA
DKFZp686J14194,FJ	FtsJ homolog 2 (E. coli	ENSG000001	UCSC	Graphic View	NA
NFAT5	Nuclear factor of activa	ENSG000001	UCSC	Graphic View	NA
DDR1	Discoidin domain recep	ENSG000002	UCSC	Graphic View	NA
PON2	Paraoxonase 2 (PON2)	ENSG000001	UCSC	Graphic View	NA
CDC25B	Cell division cycle 25B	ENSG000001	UCSC	Graphic View	NA
AD013,CHD9,CReMM	Chromodomain helicas	ENSG000001	UCSC	Graphic View	NA
FBL4,FBL5,FBXL5,FL	F-box and leucine-rich	ENSG000001	UCSC	Graphic View	NA
SCYL1	SCY1-like 1|SCY1-like	ENSG000001	UCSC	Graphic View	NA
G3BP,HDH-VIII,MGC1	Ras-GTPase-activating	ENSG000001	UCSC	Graphic View	NA
G3BP,HDH-VIII,MGC1	Ras-GTPase-activating	ENSG000001	UCSC	Graphic View	NA
EXOSC9,PM/Scl-75,F	Exosome complex exo	ENSG000001	UCSC	Graphic View	NA
LETMD1	LETM1 domain contain	ENSG000000	UCSC	Graphic View	NA
DKFZp667O0311,ME	Nuclear RNA export fac	ENSG000001	UCSC	Graphic View	NA
FLJ41118,MGC72094	Tropomyosin 3 (TPM3)	ENSG000001	UCSC	Graphic View	NA
OSBPL2	Oxysterol binding prote	ENSG000001	UCSC	Graphic View	NA
CWF19L1,FLJ10998,	CWF19-like 1, cell cycl	ENSG000000	UCSC	Graphic View	NA
RBM34	RNA-binding protein 34	ENSG000001	UCSC	Graphic View	NA
DKFZp762F117,DPP9	Dipeptidyl peptidase 9	ENSG000001	UCSC	Graphic View	NA
CEP170	Centrosomal protein 17	ENSG000001	UCSC	Graphic View	NA
FLJ12155,IPO3,KPNE	Transportin 2 (importin	ENSG000001	UCSC	Graphic View	NA
ATP13A1	ATPase type 13A1 (AT	ENSG000001	UCSC	Graphic View	NA
KIAA0595,MGC74642	PGC-1 related co-activ	ENSG000001	UCSC	Graphic View	NA
RPLP0	60S acidic ribosomal pr	ENSG000000	UCSC	Graphic View	NA
C12orf41,FLJ12670,F	Chromosome 12 open	ENSG000001	UCSC	Graphic View	NA
---	Colon carcinoma relate	ENSG000002	UCSC	Graphic View	NA
1110054L24Rik,HSPC	Nuclear-interacting par	ENSG000000	UCSC	Graphic View	NA
MBD4	Methyl-CpG binding do	ENSG000001	UCSC	Graphic View	NA
PLEKHM2	PLEKHM2 protein (Fra	ENSG000001	UCSC	Graphic View	NA
HSPA8	Heat shock 70kDa prot	ENSG000001	UCSC	Graphic View	NA
---	GPR108 protein	ENSG000001	UCSC	Graphic View	NA
ZNF561	Zinc finger protein 561|	ENSG000001	UCSC	Graphic View	NA
CDH-GAMMA-A5,CD	Protocadherin gamma	ENSG000000	UCSC	Graphic View	NA
FAM45A,FAM45B	Family with sequence s	ENSG000001	UCSC	Graphic View	probeset for
SLC39A6	Solute carrier family 39	ENSG000001	UCSC	Graphic View	probeset for
ALG9,DIBD1,DKFZp5	Alpha-1,2-mannosyltra	ENSG000000	UCSC	Graphic View	probeset for
FLJ10330,MGC41809	PRP38 pre- processing	ENSG000001	UCSC	Graphic View	probeset for
DKFZp686F1765,ILK,	Integrin-linked kinase (	ENSG000001	UCSC	Graphic View	probeset for
DKFZp686F1765,ILK,	Integrin-linked kinase (	ENSG000001	UCSC	Graphic View	probeset for
DKFZP564K247,HIG1	HIG1 domain family me	ENSG000001	UCSC	Graphic View	probeset for
DKFZP564K247,HIG1	HIG1 domain family me	ENSG000001	UCSC	Graphic View	probeset for
ALIEN,COPS2,CSN2	COP9 constitutive phot	ENSG000001	UCSC	Graphic View	probeset for
GNAS	GNAS complex locus (	ENSG000000	UCSC	Graphic View	probeset for
RNF130	Goliath homolog precu	ENSG000001	UCSC	Graphic View	probeset for
MGC2734,RBM18,RF	RNA binding motif prote	ENSG000001	UCSC	Graphic View	probeset for
ARID1B	AT rich interactive dom	ENSG000000	UCSC	Graphic View	probeset for
TOP2B,TOPIIB,top2b	DNA topoisomerase 2-(	ENSG000000	UCSC	Graphic View	probeset for
NKIRAS2	NF-kappa-B inhibitor-in	ENSG000001	UCSC	Graphic View	probeset for

FLJ12549,NUP85,Nu	Nucleoporin 85|Nucleo	ENSG000001	UCSC	Graphic View	probeset for
PRPF40A	Pre--processing factor	ENSG000001	UCSC	Graphic View	probeset for
KIAA2010,MSTP033,S	KIAA2010 (KIAA2010),	ENSG000001	UCSC	Graphic View	probeset for
PB1	Polybromo 1 (PB1), va	ENSG000001	UCSC	Graphic View	probeset for
NY-SAR-48	Sarcoma antigen NY-S	ENSG000001	UCSC	Graphic View	probeset for
M6A,METTL3,MT-A70	Methyltransferase like 3	ENSG000001	UCSC	Graphic View	probeset for
NUP160	Nuclear pore complex p	ENSG000000	UCSC	Graphic View	probeset for
ACAD-9,ACAD9,MGC	Acyl-CoA dehydrogena	ENSG000001	UCSC	Graphic View	probeset for
6530402N02Rik,APH-	Anterior pharynx defect	ENSG000001	UCSC	Graphic View	probeset for
6530402N02Rik,APH-	Anterior pharynx defect	ENSG000001	UCSC	Graphic View	probeset for
AD3,FAD,PS1,PSEN1	"Presenilin 1 (Alzheime	ENSG000000	UCSC	Graphic View	probeset for
CLONE243,CNOT4,N	CCR4-NOT ion comple	ENSG000000	UCSC	Graphic View	probeset for
CLONE243,CNOT4,N	CCR4-NOT ion comple	ENSG000000	UCSC	Graphic View	probeset for
KIAA1033	---	ENSG000001	UCSC	Graphic View	probeset for
C21orf1,C21orf3,PBF	Pituitary tumor-transfor	ENSG000001	UCSC	Graphic View	probeset for
Gp95,NT3,SORT1	Sortilin 1 (SORT1)|Sort	ENSG000001	UCSC	Graphic View	probeset for
PIAS3	Protein inhibitor of activ	ENSG000001	UCSC	Graphic View	probeset for
FLJ11021	Similar to splicing facto	ENSG000001	UCSC	Graphic View	probeset for
DRG2	Developmentally regula	ENSG000001	UCSC	Graphic View	probeset for
MORC2	MORC family CW-type	ENSG000001	UCSC	Graphic View	probeset for
C11orf51,DKFZP564M	Chromosome 11 open	ENSG000001	UCSC	Graphic View	probeset for
KDP,KIAA0344,PHA2	Serine/threonine-protei	ENSG000000	UCSC	Graphic View	probeset for
PUM2	Pumilio homolog 2 (Dro	ENSG000000	UCSC	Graphic View	probeset for
FLAD1	FAD synthetase isoform	ENSG000001	UCSC	Graphic View	probeset for
ENT4,FLJ34923,PMA	Solute carrier family 29	ENSG000001	UCSC	Graphic View	probeset for
EPLIN,LIMA1,MGC13	Epithelial protein lost in	ENSG000000	UCSC	Graphic View	probeset for
160-KD,MGC39488,P	Serine/arginine repetitiv	ENSG000001	UCSC	Graphic View	probeset for
CDKN3	Cyclin-dependent kinas	ENSG000001	UCSC	Graphic View	probeset for
C19orf48	Chromosome 19 open	ENSG000001	UCSC	Graphic View	probeset for
C19orf48	Chromosome 19 open	ENSG000001	UCSC	Graphic View	probeset for
BTBD10,GMRP-1,GM	BTB (POZ) domain con	ENSG000001	UCSC	Graphic View	probeset for
CIC	Capicua homolog (Dros	ENSG000000	UCSC	Graphic View	probeset for
COPS3	COP9 constitutive phot	ENSG000001	UCSC	Graphic View	probeset for
---	KIAA0261	ENSG000000	UCSC	Graphic View	probeset for
RNF111	Ring finger protein 111|	ENSG000001	UCSC	Graphic View	probeset for
C20orf30,HSPC274,d	Chromosome 20 open	ENSG000000	UCSC	Graphic View	probeset for
SCP2	Nonspecific lipid-transfe	ENSG000001	UCSC	Graphic View	probeset for
BAT2-iso,BAT2D1,XT	BAT2 domain containin	ENSG000001	UCSC	Graphic View	probeset for
PAPD4	PAP associated domain	ENSG000001	UCSC	Graphic View	probeset for
QTRTD1	Queuine tRNA-ribosyltr	ENSG000001	UCSC	Graphic View	probeset for
MFN1	Mitofusin 1 (MFN1), nu	ENSG000001	UCSC	Graphic View	probeset for
CALD1,CDM,H-CAD,	Caldesmon (CDM)|Cald	ENSG000001	UCSC	Graphic View	probeset for
ZNF650	Zinc finger protein 650|	ENSG000001	UCSC	Graphic View	probeset for
MTO1	Mitochondrial translatio	ENSG000001	UCSC	Graphic View	probeset for
C10orf57,FLJ13263,R	Chromosome 10 open	ENSG000001	UCSC	Graphic View	probeset for
CRMP1,DPYSL1,DRP	Collapsin response me	ENSG000000	UCSC	Graphic View	probeset for
RARSL	Arginyl-tRNA synthetas	ENSG000001	UCSC	Graphic View	probeset for
HsT17534,NEO1,NGN	Neogenin homolog 1 (d	ENSG000000	UCSC	Graphic View	low expresse
MGC104393,MGC125	NEDD8 precursor (Ubiq	ENSG000001	UCSC	Graphic View	low expresse
---	Protein raver-2	ENSG000001	UCSC	Graphic View	low expresse
CXXC9,DNMT,DNMT	DNA (cytosine-5)-meth	ENSG000001	UCSC	Graphic View	low expresse
CDC16	CDC16 cell division cyc	ENSG000001	UCSC	Graphic View	low expresse
FBX20,FBXO20,KIAA	LIM domain 7 (LMO7)|L	ENSG000001	UCSC	Graphic View	low expresse
KIAA0713,RGS-PX1,S	Sorting nexin 13 (SNX1	ENSG000000	UCSC	Graphic View	low expresse
HOMER2	Homer homolog 2 (Dro	ENSG000001	UCSC	Graphic View	low expresse
---	Pyruvate dehydrogenas	ENSG000000	UCSC	Graphic View	low expresse

SETD5	CDNA FLJ10707 fis, cl	ENSG000001	UCSC	Graphic View	ow expresse
TTC8	Tetratricopeptide repea	ENSG000001	UCSC	Graphic View	ow expresse
C8orf36,FLJ32440,MI	Non-SMC element 2 ho	ENSG000001	UCSC	Graphic View	ow expresse
MRC2	Macrophage mannose	ENSG000000	UCSC	Graphic View	ow expresse
AURKB	Aurora kinase B (AURK	ENSG000001	UCSC	Graphic View	ow expresse
FLJ11806,MGC26892	Nuclear protein UKp68	ENSG000001	UCSC	Graphic View	ow expresse
HRD,KCS,KCS1,TBC	Tubulin-specific chaper	ENSG000001	UCSC	Graphic View	ow expresse
DKFZp686J1430,SOK	Serine/threonine kinase	ENSG000001	UCSC	Graphic View	ow expresse
DKFZp686J1430,SOK	Serine/threonine kinase	ENSG000001	UCSC	Graphic View	ow expresse
DKFZp686F06131,PII	Tight junction associate	ENSG000001	UCSC	Graphic View	ow expresse
BRD9,DKFZp686L05	Bromodomain containi	ENSG000000	UCSC	Graphic View	ow expresse
IKBKB	Inhibitor of nuclear fact	ENSG000001	UCSC	Graphic View	ow expresse
TCEB2	Ion elongation factor B	ENSG000001	UCSC	Graphic View	ow expresse
KIAA1594,MGC11726	Ubiquitin carboxyl-term	ENSG000001	UCSC	Graphic View	ow expresse
CAPC,SMC4,SMC4L1	Structural maintenance	ENSG000001	UCSC	Graphic View	ow expresse
WDR74	WD-repeat protein 74 (	ENSG000001	UCSC	Graphic View	ow expresse
RC3H1	Roquin (RING finger ar	ENSG000001	UCSC	Graphic View	ow expresse
GTF2IRD1	GTF2I repeat domain c	ENSG000000	UCSC	Graphic View	ow expresse
AGAP3,CENTG3,CRA	Centaurin, gamma 3 (C	ENSG000001	UCSC	Graphic View	ow expresse
FLJ21523,MGC26225	DNA repair protein REV	ENSG000001	UCSC	Graphic View	ow expresse
TOE1	Target of EGR1, memb	ENSG000001	UCSC	Graphic View	ow expresse
COX7A2	Cytochrome c oxidase	ENSG000001	UCSC	Graphic View	ow expresse
FLJ23033,FLJ36855,I	Tubulin tyrosine ligase-	ENSG000001	UCSC	Graphic View	ow expresse
CALCOCO3,T6BP,TA	Tax1 (human T-cell leu	ENSG000001	UCSC	Graphic View	ow expresse
DEP.5,DEPDC5,DKFZ	DEP domain containing	ENSG000001	UCSC	Graphic View	ow expresse
GPATC4	G patch domain contai	ENSG000001	UCSC	Graphic View	ow expresse
SAMD4A	Sterile alpha motif dom	ENSG000000	UCSC	Graphic View	ow expresse
NUSAP1	Nucleolar and spindle a	ENSG000001	UCSC	Graphic View	ow expresse
NUSAP1	Nucleolar and spindle a	ENSG000001	UCSC	Graphic View	ow expresse
KIAA0907	---	ENSG000001	UCSC	Graphic View	ow expresse
CHPT1,CPT,CPT1	Choline phosphotransfe	ENSG000001	UCSC	Graphic View	ow expresse
DKFZp762F117,DPP9	Dipeptidyl peptidase 9	ENSG000001	UCSC	Graphic View	tion between
LAMA5	Laminin alpha-5 chain	ENSG000001	UCSC	Graphic View	tion between
CELIAC4,MYO9B,MY	Myosin IXB (MYO9B)|N	ENSG000000	UCSC	Graphic View	tion between
NIPBL	Nipped-B homolog (Dro	ENSG000001	UCSC	Graphic View	tion between
GPS2,KIAA1787	G protein pathway sup	ENSG000001	UCSC	Graphic View	tion between
---	CDNA FLJ45662 fis, cl	ENSG000001	UCSC	Graphic View	tion between
ANKHD1,EIF4EBP3,M	Ankyrin repeat and KH	ENSG000001	UCSC	Graphic View	tion between
DKFZp779A1753,MA	Microtubule-associated	ENSG000000	UCSC	Graphic View	tion between
7h3,FLJ13511,SYDE1	Synapse defective 1, R	ENSG000001	UCSC	Graphic View	tion between
BFZB,C20orf44,CBP3	Basic FGF-repressed Z	ENSG000001	UCSC	Graphic View	tion between
PRA1,PRAF1,RABAC	Prenylated Rab accept	ENSG000001	UCSC	Graphic View	tion between
BAX	Apoptosis regulator BA	ENSG000000	UCSC	Graphic View	tion between
FLJ12568,LRRTM4,M	Leucine rich repeat tra	ENSG000001	UCSC	Graphic View	tion between
RTN4	Reticulon 4 (RTN4), va	ENSG000001	UCSC	Graphic View	tion between
---	---	ENSG000001	UCSC	Graphic View	tion between
FLJ20917,MGC22737	Mitochondrial ribosoma	ENSG000001	UCSC	Graphic View	tion between
FLJ20232,HSU79252	Smith-Magenis syndro	ENSG000001	UCSC	Graphic View	tion between
AMDM,ANPRB,GUC2	Atrial natriuretic peptid	ENSG000001	UCSC	Graphic View	tion between
AUF1,AUF1A,HNRPD	Heterogeneous nuclea	ENSG000001	UCSC	Graphic View	tion between
AUF1,AUF1A,HNRPD	Heterogeneous nuclea	ENSG000001	UCSC	Graphic View	tion between
AUF1,AUF1A,HNRPD	Heterogeneous nuclea	ENSG000001	UCSC	Graphic View	tion between
AUF1,AUF1A,HNRPD	Heterogeneous nuclea	ENSG000001	UCSC	Graphic View	tion between
TSPAN17	Tetraspanin 17 (TSPAN	ENSG000000	UCSC	Graphic View	tion between
TAF6	Ion initiation factor TFII	ENSG000001	UCSC	Graphic View	tion between
KSR1,LGALS9	Galectin-9 (HOM-HD-2	ENSG000001	UCSC	Graphic View	en probesets

BMP1,FLJ44432,PCO	Bone morphogenetic pr	ENSG000001	UCSC	Graphic View	en probesets
AKT1,MGC99656,PK	RAC-alpha serine/threo	ENSG000001	UCSC	Graphic View	en probesets
ASCC2	Activating signal cointe	ENSG000001	UCSC	Graphic View	en probesets
MTA1	Metastasis associated	ENSG000001	UCSC	Graphic View	en probesets
KIAA0652	KIAA0652 (KIAA0652)	ENSG000001	UCSC	Graphic View	en probesets
EDD1	E3 ubiquitin protein liga	ENSG000001	UCSC	Graphic View	en probesets

Chromosome	Strand	Start (hg18)	End (hg18)	Probeset Name	Intensity Neg
chr3	+	171183142	171183189	JUC0300065453	959.18
chr7	-	138949698	138949779	JUC0700123025	2530.47
chr8	+	22109639	22110138	JUC0800006236	754.77
chr17	+	37974014	37974103	JUC1700036072	338.28
chr17	-	39628722	39628779	JUC1700118010	823.92
chr16	+	48816515	48816637	JUC1600035767	717.48
chr12	+	6749271	6749348	JUC1200005246	1267.36
chr1	+	2314507	2314569	JUC0100003780	647.79
chr11	-	492181	492249	JUC1100076392	569.12
chr11	-	492181	492249	JUC1100076392	569.12
chr11	-	492181	492249	JUC1100076392	569.12
chr11	-	492181	492249	JUC1100076327	493.46
chr10	-	5848463	5848607	JUC1000058166	187.34
chr4	-	140212607	140212643	JUC0400084382	844.13
chr8	+	48355121	48355158	JUC0800018564	80.98
chr15	+	37661201	37661322	JUC1500013046	1119.38
chr4	-	850289	850322	JUC0400051117	255.32
chr19	+	2222390	2222438	JUC1900004081	417.30
chr17	+	32872043	32872172	JUC1700032296	1052.62
chr5	-	157149099	157149153	JUC0500108807	1710.36
chr3	+	44383952	44384028	JUC0300017476	131.73
chr3	-	114212702	114212943	JUC0300129945	254.58
chr9	+	132563370	132563459	JUC0900052638	421.62
chr21	-	29302432	29302499	JUC2100018330	174.36
chr20	-	45300909	45301047	JUC2000053934	1432.79
chr9	-	134974893	134974929	JUC0900114152	764.17
chr3	-	51968951	51969079	JUC0300111504	136.28
chr3	-	9855669	9855716	JUC0300077613	368.54
chr1	+	44873847	44873925	JUC0100046466	2351.41
chr15	-	58547653	58547813	JUC1500087468	1799.85
chr22	-	25196779	25196888	JUC2200034180	1684.45
chr19	+	54150617	54150668	JUC1900053286	654.63
chr12	+	129923082	129923127	JUC1200071247	175.71
chr17	-	7237187	7237389	JUC1700083147	228.70
chr17	-	7237187	7237389	JUC1700083147	228.70
chr17	+	63372474	63372903	JUC1700053955	490.00
chr11	-	490654	490738	JUC1100076347	1555.31
chr9	+	108734368	108734548	JUC0900033870	1739.14
chr1	-	247117110	247117195	JUC0100299926	56.43

chr1	-	247117110	247117195	JUC0100299926	56.43
chr11	-	64290028	64290203	JUC1100108052	559.67
chr2	-	175577868	175578054	JUC0200178372	77.36
chr2	-	219232002	219232097	JUC0200257501	224.50
chr6	-	139289231	139289311	JUC0600143252	330.45
chr19	-	52609884	52609935	JUC1900121525	499.90
chr11	-	129613661	129613712	JUC1100135202	215.17
chr7	-	2245837	2245868	JUC0700071805	1766.39
chr7	-	2245837	2245868	JUC0700071805	1766.39
chr7	-	2245837	2245868	JUC0700071805	1766.39
chr16	+	68238571	68238990	JUC1600049101	141.98
chr6	+	30970262	30970344	JUC0600008227	57.20
chr7	-	94878991	94879027	JUC0700098983	333.55
chr20	+	3726269	3726311	JUC2000004107	5835.90
chr16	+	51899318	51899366	JUC1600036894	1097.70
chr4	-	15255378	15255429	JUC0400056296	94.80
chr11	+	65062014	65062051	JUC1100037952	170.79
chr5	+	151146326	151146357	JUC0500052009	428.01
chr5	+	151146326	151146357	JUC0500052009	428.01
chr4	+	122956974	122957052	JUC0400035477	1737.52
chr12	+	49733828	49733862	JUC1200023151	30.71
chr11	-	62318446	62318488	JUC1100105638	72.22
chr1	-	152415294	152415342	JUC0100249560	1398.21
chr20	+	60268432	60268468	JUC2000030711	178.40
chr10	-	102006009	102006037	JUC1000099046	88.74
chr1	-	233390449	233390585	JUC0100293321	98.19
chr19	-	4640695	4640734	JUC1900073176	1282.31
chr1	-	241386272	241386380	JUC0100297940	1372.05
chr19	-	12687524	12687553	JUC1900093070	138.65
chr19	-	19625883	19625953	JUC1900105196	1272.19
chr10	+	103888366	103888467	JUC1000038914	208.03
chr12	-	119123017	119123077	JUC1200141748	18850.78
chr12	-	47340416	47340518	JUC1200103181	927.21
chr3	-	87110483	87110642	JUC0300123161	8091.81
chr7	-	129451453	129451582	JUC0700118885	196.40
chr3	-	130634036	130634089	JUC0300138090	61.20
chr1	+	15930971	15931032	JUC0100016650	138.09
chr11	-	122435734	122435946	JUC1100132545	2640.23
chr19	-	6682245	6682293	JUC1900077588	316.56
chr19	-	9588847	9588902	JUC1900084995	1041.64
chr5	+	140838145	140838297	JUC0500047498	325.86
chr10	+	120857470	120857666	JUC1000047306	3038.26
chr18	-	31963154	31963203	JUC1800030085	1014.02
chr11	-	111214182	111214311	JUC1100127687	3756.95
chr1	+	109039847	109039915	JUC0100068421	3062.96
chr11	+	6581986	6582032	JUC1100014241	59.08
chr11	+	6581986	6582032	JUC1100014264	1927.77
chr3	-	42810653	42810771	JUC0300090700	913.45
chr3	-	42810653	42810771	JUC0300090700	913.45
chr15	-	47216837	47216926	JUC1500078817	778.59
chr20	+	56911981	56912035	JUC2000028485	8394.88
chr5	-	179323077	179323167	JUC0500116037	26.95
chr9	-	124063480	124063598	JUC0900107319	214.32
chr6	+	157495866	157496033	JUC0600062371	4334.73
chr3	-	25661795	25661950	JUC0300082890	2237.92
chr17	+	37427943	37428016	JUC1700035392	3252.26

chr17	+	70742806	70742891	JUC1700057044	94.12
chr2	-	153228636	153228727	JUC0200166446	1906.92
chr14	-	90998146	90998318	JUC1400081279	39.96
chr3	-	52688630	52688769	JUC0300112485	2785.67
chr19	-	17040841	17040896	JUC1900100692	1387.49
chr14	-	21037290	21037355	JUC1400054090	63.78
chr11	-	47791010	47791175	JUC1100099564	996.39
chr3	+	130104086	130104161	JUC0300053526	591.64
chr1	-	148507015	148507151	JUC0100244428	63.44
chr1	-	148507015	148507151	JUC0100244427	1308.49
chr14	+	72684481	72684555	JUC1400032105	406.77
chr7	-	134698261	134698378	JUC0700121172	786.24
chr7	-	134698261	134698378	JUC0700121234	1343.48
chr12	+	104039009	104039112	JUC1200053749	330.19
chr21	-	45118108	45118168	JUC2100026650	1530.74
chr1	-	109689896	109690023	JUC0100233260	1586.22
chr1	+	144289758	144289836	JUC0100078300	2005.49
chr12	-	121569339	121569529	JUC1200144549	66.68
chr17	+	17937852	17938007	JUC1700018931	37.08
chr22	-	29654015	29654194	JUC2200037468	185.85
chr11	-	71498575	71498622	JUC1100116180	4921.23
chr12	+	857642	857788	JUC1200000847	3506.37
chr2	-	20321485	20321613	JUC0200098244	4584.34
chr1	+	153229439	153229628	JUC0100085723	1892.24
chr7	+	5301022	5301091	JUC0700003377	1130.29
chr12	-	48872502	48872611	JUC1200107047	2292.29
chr1	+	24852006	24852110	JUC0100022546	4089.48
chr14	+	53954319	53954419	JUC1400014259	260.86
chr19	-	55994363	55994426	JUC1900126098	4445.55
chr19	-	55994363	55994426	JUC1900126098	4445.55
chr11	-	13399765	13399938	JUC1100086417	148.95
chr19	+	47490165	47490290	JUC1900047522	861.64
chr17	-	17120074	17120203	JUC1700094598	2862.49
chr10	-	88246938	88247038	JUC1000086942	375.04
chr15	+	57172028	57172131	JUC1500026242	362.70
chr20	-	5040146	5040251	JUC2000038867	711.56
chr1	+	53266238	53266331	JUC0100050738	330.42
chr1	+	169748764	169748935	JUC0100096271	1740.67
chr5	+	78974423	78974489	JUC0500025106	1211.68
chr3	+	115278280	115278479	JUC0300046926	351.65
chr3	+	180549330	180549445	JUC0300067168	315.78
chr7	+	134293503	134293581	JUC0700054158	1732.63
chr2	+	170571746	170571946	JUC0200054734	968.01
chr6	+	74248484	74248688	JUC0600039580	912.84
chr10	+	81831376	81831460	JUC1000028967	162.81
chr4	-	5913654	5913830	JUC0400053964	25.02
chr6	-	88286617	88286689	JUC0600119968	36.69
chr15	+	71349404	71349452	JUC1500032808	3383.48
chr14	-	23757105	23757179	JUC1400059365	4148.70
chr1	+	65043005	65043044	JUC0100055617	835.82
chr19	-	10107115	10107412	JUC1900087839	1154.36
chr13	+	114022887	114022927	JUC1300027704	616.12
chr13	+	75307289	75307370	JUC1300018002	1264.92
chr7	-	17827796	17827829	JUC0700076739	2058.56
chr15	-	81324941	81324974	JUC1500107637	1340.71
chr16	+	68733875	68733957	JUC1600050185	1195.00

chr3	+	9450961	9451018	JUC0300003705	4916.00
chr14	+	88377522	88377611	JUC1400036616	474.62
chr8	+	126183438	126183475	JUC0800038958	3143.59
chr17	+	58113149	58113328	JUC1700050283	3174.30
chr17	-	8051883	8051944	JUC1700084378	1202.34
chr14	+	88103955	88104136	JUC1400036255	1279.80
chr1	+	233649355	233649411	JUC0100138823	1061.13
chr2	-	242088884	242089165	JUC0200273362	3752.85
chr2	-	242088884	242089165	JUC0200273362	3752.85
chr6	+	43579422	43580383	JUC0600031657	470.03
chr5	-	942762	942881	JUC0500063274	1870.25
chr8	+	42270187	42270243	JUC0800017320	582.51
chr16	-	2765558	2765627	JUC1600065609	4193.45
chr2	-	219131644	219131684	JUC0200257192	875.23
chr3	+	161612935	161613331	JUC0300064937	127.16
chr11	-	62359967	62360009	JUC1100106008	641.38
chr1	-	172179349	172179376	JUC0100266852	797.15
chr7	+	73607659	73607704	JUC0700024522	675.14
chr7	+	150446494	150446525	JUC0700066808	3524.91
chr2	-	99389325	99389384	JUC0200138966	65.07
chr1	+	45580994	45581102	JUC0100047284	1026.02
chr6	-	76007559	76007612	JUC0600114870	2341.85
chr1	-	84144734	84144794	JUC0100221132	1022.09
chr7	+	27822538	27822664	JUC0700010881	2339.56
chr22	+	30562958	30562985	JUC2200013894	418.21
chr1	-	154835446	154835520	JUC0100255632	28.24
chr14	+	54320808	54320915	JUC1400014456	2844.56
chr15	+	39430481	39430523	JUC1500015849	1509.53
chr15	+	39430481	39430523	JUC1500015849	1509.53
chr1	-	154153124	154153152	JUC0100253248	455.51
chr12	+	100644313	100644406	JUC1200050093	69.90
chr19	-	4648584	4648663	JUC1900073356	30.07
chr20	-	60319867	60319987	JUC2000061716	1043.84
chr19	+	17183460	17183583	JUC1900021817	23.06
chr5	+	37099837	37099878	JUC0500008721	297.50
chr17	-	7168842	7168954	JUC1700082475	25.77
chr13	+	44842094	44842215	JUC1300013995	55.94
chr5	+	139846727	139846775	JUC0500044996	264.39
chr3	-	47887307	47887352	JUC0300094158	692.46
chr19	+	15080867	15081068	JUC1900019719	1325.15
chr20	-	33433143	33433201	JUC2000046509	16.44
chr19	-	47152882	47152926	JUC1900114680	859.01
chr19	+	54150756	54150902	JUC1900053306	785.39
chr2	-	77602382	77602452	JUC0200127506	645.91
chr2	-	55131068	55131151	JUC0200116147	218.52
chr16	-	674067	674156	JUC1600060008	425.47
chr1	-	154977428	154977469	JUC0100255812	765.44
chr22	+	38238183	38238269	JUC2200017278	726.68
chr9	+	35798506	35798680	JUC0900012381	91.75
chr4	-	83513914	83514128	JUC0400070895	2546.98
chr4	-	83513914	83514128	JUC0400070895	2546.98
chr4	-	83513914	83514128	JUC0400070895	2546.98
chr4	-	83513914	83514128	JUC0400070895	2546.98
chr5	+	176014502	176014627	JUC0500059456	19.25
chr7	-	99549175	99549239	JUC0700101228	85.34
chr17	+	22991725	22991796	JUC1700024171	23.55

chr8	+	22090382	22090419	JUC0800006470	2796.71
chr14	-	104330592	104330686	JUC1400086278	2327.58
chr22	-	28560431	28560478	JUC2200036104	2039.31
chr14	+	105001798	105001845	JUC1400049397	226.64
chr11	+	46642975	46643002	JUC1100027535	701.02
chr8	-	103380900	103380951	JUC0800072915	71.51

Junction Short Form					
Intensity 10um	Regulation SI	Fold-Change SI	P-Value SI	Probeset Name	Intensity Neg
596.04	down	1.46	1.12E-06	JUC0300065454	22.06
2558.51	up	1.22	3.90E-07	JUC0700122980	3047.72
391.50	down	1.39	9.58E-07	JUC0800006495	230.85
666.05	up	1.66	3.47E-07	JUC1700036071	174.65
783.71	down	1.52	8.47E-08	JUC1700118028	278.84
2091.13	up	2.10	1.87E-14	JUC1600035739	916.48
600.04	down	1.53	4.43E-04	JUC1200005236	357.36
548.59	down	2.06	2.72E-07	JUC0100003726	115.75
325.76	down	2.27	1.06E-08	JUC1100076328	269.84
325.76	down	2.27	1.06E-08	JUC1100076328	269.84
325.76	down	2.27	1.06E-08	JUC1100076328	269.84
177.79	down	3.69	4.95E-14	JUC1100076374	567.42
3244.57	up	13.58	8.96E-15	JUC1000058168	2148.99
581.98	down	1.23	7.44E-05	JUC0400084304	625.89
53.35	down	1.15	3.22E-03	JUC0800018524	1648.33
2445.01	up	1.13	2.60E-03	JUC1500012974	9178.57
379.69	up	1.41	7.90E-05	JUC0400050977	1606.43
6611.65	up	14.21	3.97E-16	JUC1900004085	3519.83
942.88	down	1.33	1.47E-05	JUC1700032198	350.66
328.10	down	4.87	1.56E-13	JUC0500108846	1422.74
66.95	down	1.65	5.62E-05	JUC0300017424	414.02
653.61	up	1.73	9.32E-07	JUC0300129978	2745.99
1207.40	up	2.53	6.48E-15	JUC0900052627	1626.54
207.94	up	1.76	2.10E-04	JUC2100018334	142.58
815.44	down	1.86	2.07E-11	JUC2000054084	130.40
142.78	down	6.59	2.06E-18	JUC0900114108	476.49
117.66	up	1.66	7.81E-03	JUC0300111516	617.05
205.55	down	1.24	7.12E-03	JUC0300077602	40.90
1634.23	down	1.36	1.30E-09	JUC0100046321	309.78
552.41	down	3.52	3.61E-21	JUC1500087549	596.02
1672.41	down	1.41	4.11E-07	JUC2200034254	334.50
1372.52	up	1.12	6.09E-03	JUC1900053284	1398.12
3049.48	up	13.92	2.96E-12	JUC1200071240	3834.78
415.44	up	1.53	2.87E-06	JUC1700083237	472.00
415.44	up	1.53	2.87E-06	JUC1700083237	472.00
492.32	down	1.23	4.07E-03	JUC1700054144	1170.48
1752.87	down	1.17	9.69E-05	JUC1100076321	40.68
2464.76	up	1.09	2.57E-03	JUC0900033849	846.35
31.70	down	2.68	7.84E-03	JUC0100299900	345.87

31.70	down	2.68	7.84E-03	JUC0100299900	345.87
1718.90	up	2.33	4.08E-14	JUC1100108139	6119.29
168.66	up	1.63	4.29E-05	JUC0200178480	590.99
434.05	up	1.30	1.80E-03	JUC0200257522	143.67
86.93	down	3.10	4.83E-10	JUC0600143220	4338.00
367.93	up	1.43	3.49E-05	JUC1900121577	358.49
352.84	up	1.37	3.65E-04	JUC1100135226	1540.18
1188.51	down	1.80	3.64E-11	JUC0700071796	980.57
1188.51	down	1.80	3.64E-11	JUC0700071796	980.57
1188.51	down	1.80	3.64E-11	JUC0700071796	980.57
316.39	up	1.39	2.80E-04	JUC1600048962	1472.22
80.27	up	2.07	1.96E-03	JUC0600008326	1391.98
79.07	down	4.80	1.46E-16	JUC0700099024	2176.85
6996.32	up	1.42	6.43E-06	JUC2000004124	4486.21
1178.08	down	1.14	6.28E-04	JUC1600037031	1814.08
180.35	up	1.48	1.53E-03	JUC0400056282	756.83
244.73	up	1.41	4.22E-03	JUC1100038114	2189.60
503.34	down	1.18	6.17E-05	JUC0500051957	3280.91
503.34	down	1.18	6.17E-05	JUC0500051957	3280.91
4892.58	up	2.35	3.73E-12	JUC0400035500	2254.23
39.67	up	2.10	3.44E-03	JUC1200023134	2124.66
53.25	down	1.95	6.55E-05	JUC1100105750	1440.23
1620.94	down	1.22	1.99E-04	JUC0100249245	3717.15
311.77	up	1.21	1.75E-03	JUC2000030742	1036.66
110.32	up	1.82	3.05E-04	JUC1000099117	579.30
265.48	up	2.87	2.54E-11	JUC0100293363	1421.40
954.32	up	1.32	8.46E-03	JUC1900073062	2383.34
1206.67	down	1.50	8.87E-10	JUC0100297931	2528.67
128.16	up	1.10	8.65E-03	JUC1900093247	4394.04
1666.68	down	1.43	1.38E-09	JUC1900104888	2787.72
603.84	up	1.95	4.21E-09	JUC1000038972	3820.38
22144.79	up	1.05	7.29E-03	JUC1200141738	1336.86
690.20	down	1.67	4.89E-10	JUC1200103127	3088.12
14194.38	up	1.32	1.25E-12	JUC0300123163	12386.47
343.25	up	1.41	5.33E-04	JUC0700118896	1675.71
107.79	up	1.90	6.85E-03	JUC0300138098	296.64
272.48	up	2.00	2.04E-07	JUC0100016661	2544.63
5056.43	up	1.36	1.19E-07	JUC1100132526	16288.25
352.87	up	1.67	1.11E-06	JUC1900077507	1143.33
2047.55	up	1.13	5.99E-04	JUC1900085005	61.94
453.57	up	1.26	9.59E-05	JUC0500046977	852.73
5185.06	up	1.51	1.07E-08	JUC1000047247	1093.53
540.23	down	2.53	7.36E-18	JUC1800030098	51.87
2529.42	down	1.49	1.24E-12	JUC1100127778	1767.87
1714.19	down	1.27	1.64E-03	JUC0100068409	132.99
170.27	up	4.93	1.69E-11	JUC1100014264	1927.77
852.26	down	1.32	1.51E-06	JUC1100014241	59.08
621.47	down	1.65	1.13E-08	JUC0300090699	20.47
621.47	down	1.65	1.13E-08	JUC0300090699	20.47
410.16	down	2.87	5.40E-11	JUC1500078835	27.88
12686.53	up	1.42	7.10E-11	JUC2000029034	10916.03
33.57	up	1.25	1.78E-03	JUC0500115999	400.32
610.05	up	2.19	1.66E-10	JUC0900107306	573.65
5287.49	up	1.19	1.84E-06	JUC0600062575	1121.45
2055.62	down	1.52	1.32E-05	JUC0300082767	174.37
4961.30	down	1.09	3.05E-04	JUC1700035352	63.84

214.26	up	3.05	6.28E-07	JUC1700057072	455.12
2619.19	up	1.21	8.67E-06	JUC0200166056	389.81
606.95	up	8.69	1.26E-08	JUC1400081293	876.71
1660.29	down	1.68	1.61E-08	JUC0300112614	1433.47
608.79	down	1.50	2.41E-08	JUC1900100696	156.76
69.87	up	1.94	1.46E-05	JUC1400054183	1012.59
831.94	down	1.31	1.20E-03	JUC1100100175	35.46
199.55	down	1.68	7.01E-08	JUC0300053219	52.99
259.39	up	5.58	1.13E-07	JUC0100244427	1308.49
809.85	down	1.21	4.02E-06	JUC0100244428	63.44
331.30	down	1.47	2.57E-04	JUC1400031990	1151.48
989.41	down	1.19	4.46E-03	JUC0700121234	1343.48
2509.73	up	1.24	7.41E-07	JUC0700121172	786.24
184.33	down	1.48	6.72E-04	JUC1200053990	47.31
2679.68	up	1.15	3.63E-04	JUC2100026670	161.79
1326.11	down	1.26	1.61E-06	JUC0100233365	2036.81
1096.29	down	1.32	1.61E-06	JUC0100078351	1413.38
40.39	down	2.04	6.63E-03	JUC1200144439	23.68
100.34	up	2.33	2.39E-06	JUC1700018989	1295.75
406.16	up	1.47	2.84E-06	JUC2200037594	198.15
2147.36	down	1.18	2.86E-06	JUC1100116181	118.86
2719.63	down	1.09	6.43E-05	JUC1200000531	7066.93
6117.49	down	1.10	1.67E-04	JUC0200098226	750.14
1049.38	down	1.54	6.71E-06	JUC0100085785	114.86
1036.10	up	1.56	2.16E-05	JUC0700003386	529.80
3381.52	up	1.05	5.05E-03	JUC1200107017	733.24
6067.01	down	1.21	1.50E-04	JUC0100022609	1394.13
193.38	up	1.30	2.02E-03	JUC1400014279	1874.58
3146.40	down	1.23	2.26E-03	JUC1900126107	452.53
3146.40	down	1.23	2.26E-03	JUC1900126107	452.53
183.00	up	1.62	9.22E-05	JUC1100086407	287.36
630.35	down	1.09	5.05E-03	JUC1900047475	830.62
2425.76	down	1.14	1.34E-03	JUC1700094567	160.63
620.14	up	1.23	1.98E-03	JUC1000086881	889.34
679.84	up	1.38	2.27E-04	JUC1500026252	62.56
426.85	down	1.19	4.54E-03	JUC2000038839	1769.70
396.28	up	1.63	3.67E-04	JUC0100050784	386.34
1737.86	down	1.32	5.29E-04	JUC0100096120	2378.45
1132.96	up	1.32	5.39E-04	JUC0500025058	528.78
766.90	up	1.19	7.09E-04	JUC0300046923	74.59
291.02	up	1.28	6.59E-03	JUC0300067100	1022.83
3888.41	up	1.19	1.05E-03	JUC0700054151	8221.60
874.03	down	1.20	2.14E-03	JUC0200054772	1071.03
1367.07	up	1.14	6.15E-03	JUC0600039627	107.24
109.90	down	1.25	3.13E-03	JUC1000028976	489.56
28.24	up	1.77	2.77E-03	JUC0400053924	247.10
67.38	up	1.51	2.59E-03	JUC0600120041	452.22
1959.60	down	1.51	1.89E-11	JUC1500033103	2409.90
3736.02	down	1.05	5.77E-03	JUC1400059380	89.66
400.24	down	2.48	4.44E-13	JUC0100055651	157.29
539.55	down	1.16	2.07E-04	JUC1900088378	87.78
291.05	down	1.24	6.57E-04	JUC1300027823	36.67
3561.69	up	1.47	1.34E-13	JUC1300017562	1450.08
3688.28	up	1.13	2.40E-05	JUC0700077001	91.36
270.00	down	2.58	2.63E-15	JUC1500107670	135.08
741.21	down	1.35	5.09E-06	JUC1600050093	106.73

4220.44	down	1.63	2.56E-13	JUC0300003755	465.70
1326.72	up	2.10	8.19E-14	JUC1400036527	421.54
2329.84	down	1.50	4.28E-03	JUC0800038956	168.87
2107.69	down	1.17	4.66E-05	JUC1700050180	88.26
525.26	down	1.19	5.39E-03	JUC1700084372	2862.51
1178.57	down	1.33	2.77E-06	JUC1400036423	48.32
489.21	down	1.55	7.45E-09	JUC0100138752	125.81
1441.63	down	1.34	1.26E-09	JUC0200273430	24.24
1441.63	down	1.34	1.26E-09	JUC0200273430	24.24
407.31	down	1.32	9.29E-05	JUC0600031683	135.73
1727.65	down	1.29	5.22E-05	JUC0500063172	201.16
232.71	down	1.67	5.85E-07	JUC0800017226	135.17
2420.64	down	1.13	2.67E-03	JUC1600065597	68.12
359.15	down	2.05	1.91E-10	JUC0200257276	192.35
74.91	down	1.26	8.76E-03	JUC0300064965	89.54
1029.29	up	1.12	8.25E-03	JUC1100105997	1964.01
1681.59	up	1.59	7.05E-10	JUC0100266880	279.07
165.81	down	3.86	1.19E-13	JUC0700024671	617.29
2662.35	down	1.09	9.73E-04	JUC0700066785	269.50
233.64	up	4.32	8.81E-10	JUC0200139076	836.07
875.98	down	1.45	4.33E-06	JUC0100047298	323.42
2071.94	down	1.12	4.30E-03	JUC0600114856	28.50
449.97	down	1.32	9.86E-06	JUC0100220776	81.78
2660.71	down	1.26	1.09E-04	JUC0700011044	611.40
213.78	down	1.68	7.26E-07	JUC2200013512	150.19
85.79	up	2.19	3.96E-04	JUC0100255601	247.29
3775.55	up	1.10	5.02E-04	JUC1400014427	1816.30
628.63	down	1.82	4.38E-09	JUC1500015845	2225.80
628.63	down	1.82	4.38E-09	JUC1500015845	2225.80
260.10	down	2.72	1.91E-12	JUC0100253229	1096.43
58.84	down	1.50	3.86E-03	JUC1200050128	81.42
44.91	up	2.30	2.65E-04	JUC1900073219	1597.15
1155.19	up	2.13	1.59E-04	JUC2000061216	1112.84
16.69	up	1.13	9.78E-04	JUC1900021421	83.63
114.76	down	3.32	1.36E-08	JUC0500009173	135.27
25.82	up	1.67	3.71E-04	JUC1700082435	751.92
51.59	up	1.42	4.37E-03	JUC1300013932	24.18
100.96	down	2.25	1.95E-08	JUC0500044845	2139.65
1045.88	up	1.41	2.59E-07	JUC0300094469	303.06
1228.51	down	1.74	7.96E-10	JUC1900019730	1766.62
16.58	down	1.71	6.72E-03	JUC2000046526	120.56
1052.24	up	1.54	1.81E-04	JUC1900114683	976.53
1295.61	down	1.12	1.73E-03	JUC1900053290	2534.98
594.77	up	1.29	4.22E-03	JUC0200127507	189.18
106.98	down	2.37	4.30E-07	JUC0200116209	2714.84
383.37	down	1.23	7.54E-04	JUC1600060025	2429.97
658.11	down	1.13	5.88E-03	JUC0100255791	825.28
1498.32	up	1.15	9.68E-03	JUC2200017287	749.57
136.41	up	2.29	3.74E-05	JUC0900012570	1106.39
1075.73	down	2.70	1.08E-12	JUC0400070999	2059.70
1075.73	down	2.70	1.08E-12	JUC0400070999	2059.70
1075.73	down	2.70	1.08E-12	JUC0400070999	2059.70
1075.73	down	2.70	1.08E-12	JUC0400070999	2059.70
59.92	up	3.47	3.06E-06	JUC0500059478	609.50
171.95	up	2.03	2.83E-04	JUC0700101281	1478.62
20.50	up	1.42	2.56E-03	JUC1700024247	74.41

2739.21	up	1.34	9.71E-04	JUC0800006469	472.42
2501.25	up	1.92	9.82E-05	JUC1400086204	1577.26
1802.45	down	1.09	1.12E-09	JUC2200036032	1556.85
326.24	up	1.90	7.16E-06	JUC1400049568	2349.24
561.38	down	1.97	9.48E-07	JUC1100027275	103.11
134.42	up	2.04	8.51E-03	JUC0800072074	864.71

Junction Long Form					
Intensity 10um	Regulation SI	Fold-Change SI	P-Value SI	Probeset Name	Intensity Neg
49.57	up	2.50	2.97E-05	PSR030007057	605.58
238.27	down	10.39	1.03E-18	PSR070014344	3390.05
1109.60	up	6.52	7.80E-17	PSR080000910	1305.87
144.31	down	1.42	1.91E-03	PSR170004082	4123.21
1005.33	up	2.48	1.37E-13	PSR170013538	249.42
421.39	down	3.04	2.86E-13	PSR160004387	3665.53
669.29	up	2.52	8.33E-12	PSR120000581	3706.96
402.59	up	1.98	1.13E-05	PSR010000433	555.29
1220.30	up	3.42	7.92E-16	PSR110009002	1182.07
1220.30	up	3.42	7.92E-16	PSR110009002	1182.07
1220.30	up	3.42	7.92E-16	PSR110009002	1182.07
1324.11	up	1.77	1.74E-10	PSR110009002	1182.07
1270.95	down	2.17	1.17E-11	PSR100007084	2810.87
820.60	up	1.57	6.64E-07	PSR040010237	1992.06
2406.23	up	1.94	8.61E-09	PSR080002271	728.94
10954.66	down	1.61	1.39E-06	PSR150001078	2166.45
1385.40	down	1.22	3.44E-04	PSR040005986	743.29
1940.56	down	1.96	4.97E-11	PSR190000571	10055.92
690.20	up	1.66	1.44E-07	PSR170003423	1657.35
2287.90	up	1.70	7.64E-11	PSR050012372	2860.68
666.53	up	1.93	7.48E-11	PSR030002030	156.38
2116.52	down	1.90	2.10E-10	PSR030013557	1150.13
1023.67	down	1.77	4.85E-12	PSR090005582	2235.75
50.78	down	1.95	5.16E-04	PSR210002076	1268.59
289.55	up	2.13	1.52E-08	PSR200007218	1326.78
970.32	up	1.64	1.11E-08	PSR090012739	750.09
202.10	down	1.59	3.11E-06	PSR030011622	2615.97
30.59	up	1.12	6.58E-03	PSR030008722	986.45
476.92	up	1.61	5.99E-05	PSR010004961	2179.42
1096.38	up	1.67	4.28E-08	PSR150008579	1663.28
884.28	up	1.87	8.92E-12	PSR220004674	1944.76
1244.48	down	2.07	1.76E-08	PSR190006919	1740.62
3176.34	down	1.49	4.90E-09	PSR120007707	14946.67
279.82	down	1.97	5.57E-12	PSR170009492	367.04
279.82	down	1.97	5.57E-12	PSR170009492	367.04
1797.53	up	1.24	1.30E-04	PSR170006408	6275.07
107.22	up	2.09	2.39E-04	PSR110009000	1682.02
811.01	down	1.35	5.02E-06	PSR090003742	2443.16
800.89	up	1.48	2.47E-06	PSR010033511	49.68

800.89	up	1.48	2.47E-06	PSR010033511	49.68
4545.78	down	1.75	5.76E-13	PSR110013105	3170.40
470.38	down	1.67	1.84E-08	PSR020018798	1672.31
156.10	down	1.38	8.02E-03	PSR020021003	280.55
4539.32	up	1.26	2.58E-10	PSR060015037	5275.07
164.58	down	1.11	9.80E-05	PSR190015665	401.01
1422.54	down	1.29	9.64E-07	PSR110017014	1227.09
1448.54	up	1.22	1.02E-03	PSR070008025	971.00
1448.54	up	1.22	1.02E-03	PSR070008025	971.00
1448.54	up	1.22	1.02E-03	PSR070008025	971.00
2105.32	down	1.13	3.31E-03	PSR160005915	520.25
769.95	down	1.24	1.36E-03	PSR060001558	395.63
2930.59	up	1.18	6.92E-05	PSR070011826	1435.87
3478.92	down	1.11	2.00E-03	PSR200000421	6802.51
2629.94	up	1.19	1.97E-04	PSR160004521	1757.53
407.82	down	2.40	1.35E-08	PSR040006725	1554.74
1894.32	down	1.19	2.72E-05	PSR110004554	649.10
5309.91	up	1.15	3.65E-06	PSR050005545	2982.99
5309.91	up	1.15	3.65E-06	PSR050005545	2982.99
2290.11	down	1.16	7.55E-04	PSR040004160	4326.25
887.72	down	1.50	2.43E-09	PSR120002865	2150.15
2413.60	up	1.16	4.98E-04	PSR110012745	1099.95
5661.26	up	1.07	4.41E-03	PSR010027547	4325.27
1310.63	down	1.15	1.78E-03	PSR200003893	1039.37
342.05	down	1.20	6.10E-03	PSR100011600	936.62
1071.28	down	1.30	6.08E-03	PSR010032867	650.83
1304.85	down	1.09	3.21E-03	PSR190009983	691.99
3766.96	up	1.13	1.27E-04	PSR010033215	2303.32
2690.53	down	1.36	1.13E-09	PSR190011782	2998.51
5617.32	up	1.08	8.27E-03	PSR190013128	3791.94
4780.71	down	1.19	1.52E-03	PSR100004906	2533.92
1259.40	down	1.19	2.29E-04	PSR120015499	14904.92
4314.21	up	1.12	2.77E-05	PSR120010896	7316.40
15499.84	down	1.06	5.89E-03	PSR030012908	12430.25
1802.62	down	1.15	4.25E-03	PSR070013862	846.57
192.17	down	1.43	8.83E-04	PSR030014599	751.41
2192.61	down	1.13	7.63E-03	PSR010001751	1341.24
19784.02	down	1.15	2.53E-06	PSR110016586	7556.32
531.83	down	1.45	1.38E-06	PSR190010430	970.28
39.69	down	2.72	2.32E-06	PSR190010987	1533.29
756.49	down	1.25	4.99E-04	PSR050004957	1130.87
253.99	down	4.77	1.17E-18	PSR100005909	1833.84
1404.43	up	19.87	5.16E-16	PSR180003445	781.67
2856.86	up	1.62	8.55E-10	PSR110015837	5843.26
424.06	up	4.44	3.98E-12	PSR010007874	762.94
852.26	down	1.32	1.51E-06	PSR110001163	169.82
170.27	up	4.93	1.69E-11	PSR110001163	169.82
142.08	up	6.01	2.73E-11	PSR030010072	870.98
142.08	up	6.01	2.73E-11	PSR030010072	870.98
63.87	up	1.47	9.07E-03	PSR150007792	1617.33
10641.40	down	1.10	2.86E-03	PSR200003681	11919.99
196.74	down	2.06	1.96E-09	PSR050013394	962.38
620.03	down	1.20	1.66E-03	PSR090011768	1762.63
708.46	down	1.64	6.65E-10	PSR060007255	1483.60
488.60	up	2.02	1.67E-09	PSR030009410	945.20
586.70	up	5.56	2.75E-09	PSR170003976	3439.46

195.91	down	1.74	3.10E-09	PSR170006816	651.80
245.69	down	1.81	1.20E-08	PSR020017741	1176.33
1291.01	down	1.18	3.40E-03	PSR140008644	576.48
1872.02	up	1.30	1.42E-03	PSR030011800	1300.60
176.35	up	1.69	3.35E-04	PSR190012585	1964.04
403.91	down	1.42	4.94E-08	PSR140005312	1229.64
129.03	up	3.28	5.34E-08	PSR110011899	1402.79
76.53	up	2.56	5.16E-05	PSR030005503	1520.60
809.85	down	1.21	4.02E-06	PSR010026732	1292.40
259.39	up	5.58	1.13E-07	PSR010026732	1292.40
1753.15	up	1.28	3.69E-07	PSR140002841	3830.34
2509.73	up	1.24	7.41E-07	PSR070014113	2503.87
989.41	down	1.19	4.46E-03	PSR070014113	2503.87
98.51	up	2.53	1.15E-06	PSR120005891	1558.54
144.84	down	1.69	1.04E-04	PSR210003209	2919.24
2471.28	up	1.15	5.99E-06	PSR010025060	2997.29
1232.26	up	1.22	4.87E-05	PSR010009392	3198.20
123.35	up	4.28	2.21E-06	PSR120015836	1648.18
1351.07	down	1.14	5.37E-04	PSR170001875	1298.79
227.50	down	1.28	5.11E-03	PSR220005087	414.59
113.96	up	1.84	5.60E-03	PSR110014226	8538.16
7297.36	up	1.23	3.72E-06	PSR120000080	4632.37
1633.28	up	1.49	5.72E-06	PSR020011758	5155.40
176.49	up	1.91	3.02E-04	PSR010010605	1065.95
255.42	down	1.25	3.65E-04	PSR070000406	1859.07
834.75	down	1.23	3.07E-05	PSR120011320	2278.12
3111.18	up	1.24	3.88E-05	PSR010002620	2711.08
904.44	down	1.19	5.89E-05	PSR140001660	1104.31
667.36	up	1.71	6.01E-05	PSR190016471	1504.96
667.36	up	1.71	6.01E-05	PSR190016471	1504.96
166.10	down	1.31	7.92E-03	PSR110010441	596.64
772.87	up	1.19	9.79E-05	PSR190005889	1284.88
252.89	up	1.65	1.18E-04	PSR170010510	2732.35
994.81	down	1.20	1.72E-04	PSR100010536	1613.29
30.86	down	2.74	3.02E-04	PSR150002528	609.77
2234.43	up	1.80	2.11E-03	PSR200005001	3066.68
239.19	down	1.19	2.75E-04	PSR010005632	301.12
3656.55	up	1.17	4.75E-03	PSR010012305	1563.63
246.08	down	1.50	1.61E-03	PSR050002642	2486.90
86.48	down	1.54	5.36E-03	PSR030004817	965.27
620.82	down	1.19	7.17E-04	PSR030007284	404.94
14364.04	down	1.08	7.01E-03	PSR070006208	7032.52
1434.14	up	1.23	1.14E-03	PSR020006941	2803.82
91.72	down	1.55	2.17E-03	PSR060004698	1646.48
509.50	up	1.25	2.24E-03	PSR100003437	211.19
118.38	down	1.33	2.31E-03	PSR040006405	898.20
446.12	down	1.23	2.60E-03	PSR060012899	786.07
3345.67	up	1.59	2.09E-07	PSR150003349	2553.45
196.85	up	2.34	1.16E-08	PSR140005874	3006.85
1411.16	up	7.73	3.14E-14	PSR010006195	1678.83
119.77	up	2.57	3.62E-03	PSR190011132	2236.19
54.19	up	2.42	4.64E-03	PSR130002811	1422.95
833.22	down	3.32	4.18E-18	PSR130001802	813.99
69.66	down	2.02	3.63E-05	PSR070008713	1254.86
289.96	up	4.17	6.46E-12	PSR150010486	2355.62
195.39	up	2.26	5.48E-06	PSR160006018	3859.84

2042.93	up	3.16	4.31E-10	PSR030000288	7512.03
170.20	down	3.33	1.43E-12	PSR140003464	1139.31
449.49	up	2.36	4.81E-10	PSR080004330	861.00
221.46	up	3.15	1.85E-10	PSR170006024	3274.89
3505.68	up	2.41	8.19E-03	PSR170009701	2276.39
109.83	up	1.87	1.80E-05	PSR140003433	2164.16
203.93	up	2.34	3.58E-06	PSR010016097	720.74
20.18	up	1.61	3.54E-03	PSR020022353	3737.79
20.18	up	1.61	3.54E-03	PSR020022353	3737.79
230.92	up	1.49	8.29E-03	PSR060003716	683.66
348.64	up	1.43	5.70E-03	PSR050007054	3513.58
164.43	up	1.82	4.97E-04	PSR080002098	920.64
76.74	up	1.72	7.43E-03	PSR160008192	4856.72
293.95	up	1.82	8.65E-06	PSR020020983	420.68
163.99	up	2.45	2.55E-04	PSR030006911	1817.52
1478.50	down	1.86	9.37E-09	PSR110012793	3804.26
192.07	down	1.96	5.06E-07	PSR010029805	1336.16
1020.54	up	1.80	1.27E-09	PSR070002943	1073.46
868.28	up	3.95	2.87E-14	PSR070007437	1791.12
319.35	down	2.24	1.50E-11	PSR020015878	717.77
531.78	up	1.35	1.47E-04	PSR010005115	1053.38
53.69	up	1.84	8.24E-04	PSR060012462	2747.66
113.79	up	2.43	7.16E-06	PSR010023750	1428.88
1071.70	up	1.22	4.71E-04	PSR070001164	1780.18
219.16	up	1.76	6.14E-04	PSR220001809	278.54
269.35	down	1.28	3.75E-03	PSR010028314	485.79
1276.30	down	1.72	2.30E-09	PSR140001689	1707.17
1978.30	up	1.17	1.33E-03	PSR150001384	3359.27
1978.30	up	1.17	1.33E-03	PSR150001384	3359.27
2057.26	up	1.19	5.56E-05	PSR010028039	1044.13
163.63	up	1.56	7.86E-05	PSR120005671	2812.68
1462.07	up	1.57	1.45E-07	PSR190009989	1651.43
1397.13	up	2.42	1.79E-04	PSR200007881	644.98
95.63	up	1.79	3.91E-03	PSR190002987	894.02
75.66	down	2.29	1.28E-05	PSR050000958	536.49
814.46	up	1.79	4.06E-09	PSR170009476	506.80
22.48	up	1.33	9.32E-04	PSR130001211	436.96
1393.93	down	1.33	5.78E-08	PSR050004729	560.60
430.67	up	1.32	4.40E-04	PSR030010495	3619.68
1873.95	down	1.52	1.30E-06	PSR190002694	510.86
86.88	down	2.40	3.33E-06	PSR200006210	233.04
685.14	down	1.13	8.01E-03	PSR190014748	946.14
3691.13	down	1.27	2.26E-04	PSR190006917	1194.82
62.60	down	2.19	2.03E-09	PSR020014740	1883.74
4158.92	up	1.32	7.67E-09	PSR020013542	1350.51
3349.24	up	1.24	1.10E-03	PSR160007495	1543.83
948.82	up	1.20	2.35E-03	PSR010028365	765.42
1105.91	down	1.21	3.89E-03	PSR220002427	1497.53
599.05	down	1.16	3.58E-03	PSR090001335	1749.83
5370.10	up	2.29	1.51E-11	PSR040008657	13246.80
5370.10	up	2.29	1.51E-11	PSR040008657	13246.80
5370.10	up	2.29	1.51E-11	PSR040008657	13246.80
5370.10	up	2.29	1.51E-11	PSR040008657	13246.80
473.43	down	1.14	2.24E-03	PSR050006456	2183.41
1086.73	down	1.38	2.70E-06	PSR070012203	2129.97
124.14	up	2.66	6.54E-07	PSR170002453	2349.88

469.87	up	1.36	1.84E-03	PSR080000898	1011.93
1388.24	up	1.58	2.32E-06	PSR140009400	1059.28
1440.13	down	1.03	6.46E-05	PSR220004937	903.32
2692.90	up	1.50	1.90E-09	PSR140004863	2263.97
255.73	up	1.56	2.78E-05	PSR110002851	731.67
560.12	down	1.38	2.87E-07	PSR080008576	510.45

Exon Short Form					
Intensity 10um	Regulation SI	Fold-Change SI	P-Value SI	Probeset Name	Intensity Neg
545.94	up	1.01	4.32E-01	PSR030007056	81.59
2478.94	down	1.13	1.60E-03	PSR070014345	3442.87
840.01	down	1.13	4.49E-03	PSR080000909	507.37
4661.87	down	1.04	2.07E-02	PSR170004080	755.88
303.89	down	1.17	2.06E-01	PSR170013539	205.16
4328.43	down	1.18	3.96E-02	PSR160004386	559.67
2168.01	down	1.23	5.40E-08	PSR120000580	529.29
1102.62	up	1.14	4.02E-02	PSR010000432	315.10
1765.47	up	1.14	6.03E-04	PSR110009003	998.20
1765.47	up	1.14	6.03E-04	PSR110009003	998.20
1765.47	up	1.14	6.03E-04	PSR110009003	998.20
1765.47	up	1.14	6.03E-04	PSR110009003	998.20
4152.78	up	1.16	1.82E-06	PSR100007086	3808.26
2010.09	up	1.20	5.51E-04	PSR040010238	667.01
1132.97	up	2.06	1.39E-09	PSR080002272	458.73
4595.04	up	1.09	1.07E-02	PSR150001077	514.75
810.13	up	1.02	2.07E-01	PSR040005987	737.52
10262.39	down	1.06	1.32E-02	PSR190000568	5243.59
1634.29	down	1.21	4.54E-03	PSR170003424	366.40
2976.72	up	1.10	1.36E-01	PSR050012373	1446.82
150.30	up	1.17	1.75E-01	PSR030002029	428.19
1666.60	up	1.00	4.36E-01	PSR030013558	2360.65
2233.18	down	1.13	1.52E-03	PSR090005584	1152.25
1409.65	up	1.61	1.33E-08	PSR210002074	822.25
1275.02	down	1.09	3.34E-03	PSR200007217	667.93
776.57	down	1.19	1.77E-02	PSR090012740	412.01
1542.03	up	1.13	1.11E-02	PSR030011620	1692.72
713.62	up	1.04	1.50E-02	PSR030008721	674.53
2242.60	up	1.08	6.12E-02	PSR010004962	849.37
1920.46	up	1.06	1.12E-03	PSR150008580	663.61
2154.00	down	1.27	3.77E-06	PSR220004675	535.50
2844.16	down	1.13	1.91E-02	PSR190006917	1194.82
17945.89	down	1.02	2.85E-01	PSR120007705	4277.39
678.86	up	1.55	3.98E-05	PSR170009490	554.55
678.86	up	1.55	3.98E-05	PSR170009490	554.55
8400.47	up	1.09	1.78E-03	PSR170006409	1520.07
2543.50	up	1.16	4.63E-05	PSR110009001	570.11
3245.05	up	1.03	3.50E-01	PSR090003741	1204.76
54.55	down	1.43	2.25E-01	PSR010033510	635.63

54.55	down	1.43	2.25E-01	PSR010033510	635.63
4160.16	up	1.01	5.94E-01	PSR110013108	2491.23
2094.72	down	1.07	4.91E-01	PSR020018796	587.60
345.69	down	1.21	4.70E-02	PSR020021004	618.84
4146.63	down	1.06	8.46E-04	PSR060015035	3166.95
264.56	up	1.29	8.53E-02	PSR190015666	1129.72
1474.35	up	1.02	3.44E-01	PSR110017015	644.41
1098.75	down	1.07	9.60E-02	PSR070008026	654.94
1098.75	down	1.07	9.60E-02	PSR070008026	654.94
1098.75	down	1.07	9.60E-02	PSR070008026	654.94
757.82	down	1.09	1.10E-01	PSR160005916	1133.49
282.73	up	1.08	3.32E-01	PSR060001557	812.17
1600.42	down	1.02	8.79E-02	PSR070011827	451.68
5973.11	up	1.03	3.62E-01	PSR200000420	1105.96
2199.88	up	1.03	2.31E-01	PSR160004522	1487.81
1869.29	down	1.07	1.66E-01	PSR040006726	2057.53
598.36	down	1.12	2.16E-02	PSR110004553	2510.83
4242.67	up	1.02	2.12E-01	PSR050005544	424.50
4242.67	up	1.02	2.12E-01	PSR050005544	424.50
5074.05	down	1.01	2.52E-01	PSR040004164	2557.29
1089.98	down	1.22	5.16E-06	PSR120002864	1939.42
1548.73	down	1.03	4.77E-01	PSR110012746	1411.59
6692.08	up	1.10	4.34E-03	PSR010027548	2796.11
1472.73	down	1.03	4.25E-01	PSR200003892	654.94
653.29	down	1.01	2.64E-02	PSR100011599	1192.23
624.15	down	1.00	5.02E-01	PSR010032865	997.38
401.68	down	1.02	2.16E-01	PSR190009984	2212.49
3121.59	up	1.03	1.30E-01	PSR010033216	3229.89
2394.67	down	1.04	3.98E-03	PSR190011783	3367.46
6916.78	down	1.03	4.56E-01	PSR190013129	1356.21
3658.29	down	1.03	2.65E-01	PSR100004905	2955.24
16946.16	up	1.02	4.27E-01	PSR120015500	13466.78
9003.58	down	1.01	2.68E-01	PSR120010895	4336.19
16299.91	down	1.00	6.05E-01	PSR030012909	10012.00
1039.43	down	1.01	2.07E-01	PSR070013863	1327.07
693.40	up	1.01	5.35E-01	PSR030014598	1610.07
1110.20	down	1.18	1.65E-03	PSR010001750	1771.83
11147.03	up	1.06	5.13E-03	PSR110016588	13445.52
569.59	down	1.12	4.57E-02	PSR190010431	1782.69
2803.74	up	1.06	2.40E-03	PSR190010988	367.93
1387.21	up	1.10	6.01E-02	PSR050004962	2486.36
2243.36	up	1.09	9.86E-03	NA	NA
953.89	down	1.10	4.67E-02	NA	NA
5797.99	up	1.00	1.60E-01	NA	NA
598.26	up	1.10	1.78E-01	NA	NA
154.45	up	1.56	4.12E-03	NA	NA
154.45	up	1.56	4.12E-03	NA	NA
927.40	down	1.06	1.87E-01	NA	NA
927.40	down	1.06	1.87E-01	NA	NA
2667.36	up	1.09	2.00E-01	NA	NA
11867.89	down	1.07	4.83E-02	NA	NA
644.75	down	1.49	8.43E-11	NA	NA
2546.03	up	1.11	7.70E-03	NA	NA
1123.07	down	1.37	2.61E-05	NA	NA
1206.71	down	1.09	3.64E-02	NA	NA
5643.28	down	1.02	2.43E-01	NA	NA

519.77	up	1.06	2.28E-01	NA	NA
1348.16	up	1.00	4.13E-01	NA	NA
1113.37	up	1.10	3.54E-02	NA	NA
1398.52	up	1.06	6.92E-02	NA	NA
1337.24	up	1.04	3.12E-01	NA	NA
736.99	up	1.06	1.09E-02	NA	NA
1372.04	down	1.12	4.77E-03	NA	NA
893.28	up	1.05	7.56E-02	NA	NA
1014.73	up	1.06	1.78E-02	NA	NA
1014.73	up	1.06	1.78E-02	NA	NA
4397.25	down	1.03	2.28E-01	NA	NA
3761.57	down	1.01	3.85E-02	NA	NA
3761.57	down	1.01	3.85E-02	NA	NA
1170.80	down	1.10	2.28E-02	NA	NA
2837.31	down	1.55	1.30E-06	NA	NA
3184.63	up	1.01	6.54E-01	NA	NA
2397.27	up	1.05	1.54E-01	NA	NA
2048.86	up	1.02	4.40E-01	NA	NA
1546.02	up	1.02	3.41E-01	NA	NA
570.04	down	1.08	3.31E-02	NA	NA
4427.85	up	1.00	5.04E-01	NA	NA
3733.56	down	1.05	1.23E-02	NA	NA
7496.99	down	1.01	4.98E-01	NA	NA
994.64	up	1.13	1.46E-01	NA	NA
1023.58	down	1.08	9.55E-03	NA	NA
3171.35	down	1.01	4.02E-01	NA	NA
5332.74	up	1.10	3.46E-04	NA	NA
719.77	up	1.14	8.84E-02	NA	NA
1148.63	down	1.13	2.15E-02	NA	NA
1148.63	down	1.13	2.15E-02	NA	NA
607.11	up	1.34	5.01E-04	NA	NA
1063.34	up	1.05	2.36E-01	NA	NA
2477.76	down	1.06	5.54E-02	NA	NA
2218.34	up	1.02	6.63E-01	NA	NA
971.83	up	1.18	2.61E-03	NA	NA
3857.60	up	1.81	2.52E-04	NA	NA
194.06	down	1.15	7.65E-03	NA	NA
1984.97	down	1.03	5.27E-01	NA	NA
1758.81	down	1.00	5.15E-01	NA	NA
1843.44	up	1.04	1.76E-02	NA	NA
327.05	up	1.11	2.55E-01	NA	NA
12818.28	down	1.04	5.64E-01	NA	NA
3016.83	down	1.02	2.57E-01	NA	NA
2090.90	down	1.04	2.81E-01	NA	NA
153.27	down	1.15	7.29E-02	NA	NA
603.18	up	1.04	3.68E-01	NA	NA
1072.16	up	1.12	3.97E-02	NA	NA
2256.43	up	1.00	4.44E-01	PSR150003348	74.65
2771.94	down	1.03	2.54E-01	PSR140005873	92.05
2114.17	up	1.06	6.19E-01	PSR010006194	30.72
1255.64	up	1.02	3.84E-01	PSR190011131	85.59
855.86	up	1.03	4.29E-02	PSR130002810	147.01
1952.24	up	1.26	3.57E-04	PSR130001801	344.29
2316.49	up	1.15	1.04E-03	PSR070008714	232.34
1776.30	up	1.47	2.36E-02	PSR150010487	98.24
3155.68	down	1.01	1.37E-01	PSR160006017	120.02

11067.75	up	1.05	2.74E-01	PSR030000287	86.63
1578.34	up	1.05	2.98E-02	PSR140003466	205.96
950.12	down	1.01	2.01E-01	PSR080004329	130.76
2559.44	up	1.01	1.35E-01	PSR170006023	178.49
993.30	down	1.17	1.49E-04	PSR170009702	168.94
2167.94	down	1.22	5.58E-04	PSR140003432	89.21
390.21	down	1.30	2.69E-05	PSR010016096	45.04
2604.34	up	1.40	9.46E-06	PSR020022354	183.42
2604.34	up	1.40	9.46E-06	PSR020022354	183.42
719.05	down	1.10	8.58E-02	PSR060003717	79.47
4350.38	up	1.04	3.07E-01	PSR050007055	169.89
456.08	down	1.34	2.51E-05	PSR080002099	136.25
3107.74	down	1.01	1.05E-02	PSR160008193	62.50
321.52	down	1.08	2.60E-01	PSR020020984	122.33
1178.13	down	1.15	1.43E-03	PSR030006912	83.05
5504.08	up	1.02	9.93E-02	PSR110012792	355.10
1707.45	down	1.03	3.18E-01	PSR010029806	310.27
679.28	down	1.45	7.67E-08	PSR070002942	246.98
1696.11	up	1.15	4.38E-04	PSR070007436	379.33
610.95	up	1.01	4.39E-01	PSR020015879	197.94
1134.93	down	1.13	9.29E-02	PSR010005114	192.82
2785.87	up	1.02	4.64E-01	PSR060012461	25.98
788.50	down	1.05	2.15E-01	PSR010023751	117.78
2160.47	down	1.16	4.89E-03	PSR070001165	203.70
233.25	down	1.04	1.15E-01	PSR220001808	213.16
709.45	up	1.04	2.65E-01	PSR010028315	343.96
2402.03	up	1.16	8.05E-05	PSR140001687	176.75
2621.43	up	1.02	7.93E-02	PSR150001383	164.32
2621.43	up	1.02	7.93E-02	PSR150001383	164.32
1616.64	down	1.00	4.95E-01	PSR010028040	110.13
3626.38	up	1.00	2.12E-01	PSR120005672	106.47
1743.10	up	1.80	2.61E-09	NA	NA
364.71	up	1.08	5.65E-01	NA	NA
790.73	up	1.39	3.66E-02	NA	NA
573.08	down	1.20	5.88E-03	PSR050000959	322.42
488.11	up	1.61	5.21E-06	PSR170009475	807.63
325.28	up	1.07	5.21E-02	PSR130001210	1740.88
491.76	up	1.02	4.63E-01	PSR050004728	2179.14
3480.85	down	1.13	4.72E-03	PSR030010494	5448.17
997.38	up	1.21	1.34E-04	PSR190002693	485.68
329.52	down	1.22	4.19E-02	PSR200006209	887.70
831.73	up	1.13	9.94E-02	PSR190014749	1270.43
1516.47	down	1.47	4.31E-06	PSR190006919	1740.62
1467.07	up	1.08	8.78E-02	PSR020014741	2502.76
1564.16	down	1.00	5.41E-01	PSR020013544	1199.47
1712.95	down	1.02	6.31E-01	PSR160007497	965.29
850.66	up	1.14	2.23E-02	PSR010028364	1086.14
2972.26	up	1.11	1.45E-05	PSR220002426	342.20
1284.00	up	1.17	1.53E-02	PSR090001333	831.59
14963.71	down	1.01	7.50E-02	PSR040008658	3850.70
14963.71	down	1.01	7.50E-02	PSR040008658	3850.70
14963.71	down	1.01	7.50E-02	PSR040008658	3850.70
14963.71	down	1.01	7.50E-02	PSR040008658	3850.70
1881.56	down	1.01	1.84E-01	PSR050006458	797.19
2041.99	down	1.06	4.44E-02	PSR070012202	3510.64
2960.80	up	2.02	4.17E-04	PSR170002452	117.00

939.81	up	1.27	1.89E-01	PSR080000897	18.13
344.57	down	1.78	1.69E-03	PSR140009401	297.79
772.46	down	1.13	2.50E-02	PSR220004936	140.12
1623.70	down	1.06	1.45E-01	PSR140004862	427.74
940.09	down	1.24	5.52E-03	PSR110002850	335.38
478.42	up	1.07	1.04E-01	PSR080008577	401.47

Exon Long Form			
Intensity 10um	Regulation SI	Fold-Change SI	P-Value SI
1056.36	up	14.38	2.05E-07
403.35	down	6.99	1.03E-13
1882.47	up	5.07	3.38E-14
333.90	down	2.66	4.61E-10
782.13	up	2.65	7.68E-08
313.96	down	2.49	6.94E-08
906.47	up	2.30	1.50E-12
1132.36	up	2.08	1.04E-10
2695.83	up	2.05	1.01E-14
2695.83	up	2.05	1.01E-14
2695.83	up	2.05	1.01E-14
2695.83	up	2.05	1.01E-14
2417.07	down	2.02	1.51E-14
1088.93	up	1.93	2.03E-08
656.29	up	1.91	1.04E-08
527.97	down	1.90	2.89E-07
417.13	down	1.85	6.38E-11
3279.47	down	1.75	2.48E-13
748.83	up	1.73	2.26E-05
2333.09	up	1.72	2.70E-10
608.53	up	1.72	1.09E-04
2038.29	down	1.70	2.95E-09
763.09	down	1.69	3.24E-10
343.10	down	1.68	1.62E-07
1159.96	up	1.66	6.95E-10
824.55	up	1.62	4.83E-08
552.70	down	1.61	2.19E-08
693.86	up	1.58	6.37E-04
1222.61	up	1.52	8.03E-08
1077.71	up	1.49	1.37E-10
1112.55	up	1.47	3.06E-07
1516.47	down	1.47	4.31E-06
3687.83	down	1.43	1.00E-08
457.27	down	1.41	1.97E-06
457.27	down	1.41	1.97E-06
2567.03	up	1.37	2.59E-08
994.32	up	1.37	5.43E-07
1142.26	down	1.37	2.21E-06
1351.88	up	1.36	5.51E-06

1351.88	up	1.36	5.51E-06
2408.45	down	1.35	1.26E-08
579.92	down	1.35	6.83E-04
698.33	down	1.33	2.74E-04
3409.98	up	1.30	4.00E-10
439.16	down	1.30	4.03E-08
604.74	down	1.28	5.43E-07
1003.54	up	1.26	2.27E-04
1003.54	up	1.26	2.27E-04
1003.54	up	1.26	2.27E-04
1494.78	down	1.21	5.33E-04
463.80	down	1.19	3.15E-02
607.73	up	1.18	2.70E-02
800.33	down	1.17	3.01E-03
2118.46	up	1.17	1.91E-04
2275.74	down	1.16	1.26E-02
2231.23	down	1.16	1.27E-05
677.51	up	1.15	1.87E-01
677.51	up	1.15	1.87E-01
2683.97	down	1.13	1.07E-02
1068.87	down	1.13	6.55E-03
2285.05	up	1.13	1.42E-02
4472.16	up	1.12	3.11E-04
845.52	down	1.12	3.56E-02
764.18	down	1.11	9.86E-02
884.17	down	1.10	1.97E-02
1208.54	down	1.09	3.53E-02
4574.57	up	1.08	1.88E-02
2579.36	down	1.08	4.57E-03
2751.36	up	1.08	2.87E-02
4050.73	down	1.08	7.79E-03
14156.94	down	1.06	4.12E-03
5611.13	up	1.05	1.66E-01
12655.78	down	1.04	6.81E-01
1574.51	down	1.04	5.90E-02
1444.98	down	1.03	5.17E-01
1702.08	down	1.02	2.65E-01
18641.79	down	1.01	3.42E-02
1183.32	down	1.01	7.62E-02
633.44	down	1.01	5.00E-01
2735.80	down	1.00	2.95E-01
NA	NA	NA	NA
NA	NA	NA	NA
NA	NA	NA	NA
NA	NA	NA	NA
NA	NA	NA	NA
NA	NA	NA	NA
NA	NA	NA	NA
NA	NA	NA	NA
NA	NA	NA	NA
NA	NA	NA	NA
NA	NA	NA	NA
NA	NA	NA	NA
NA	NA	NA	NA
NA	NA	NA	NA
NA	NA	NA	NA

NA	NA	NA	NA
NA	NA	NA	NA
NA	NA	NA	NA
NA	NA	NA	NA
NA	NA	NA	NA
NA	NA	NA	NA
NA	NA	NA	NA
NA	NA	NA	NA
NA	NA	NA	NA
NA	NA	NA	NA
NA	NA	NA	NA
NA	NA	NA	NA
NA	NA	NA	NA
NA	NA	NA	NA
NA	NA	NA	NA
NA	NA	NA	NA
NA	NA	NA	NA
NA	NA	NA	NA
NA	NA	NA	NA
NA	NA	NA	NA
NA	NA	NA	NA
NA	NA	NA	NA
NA	NA	NA	NA
NA	NA	NA	NA
NA	NA	NA	NA
NA	NA	NA	NA
NA	NA	NA	NA
NA	NA	NA	NA
NA	NA	NA	NA
NA	NA	NA	NA
NA	NA	NA	NA
NA	NA	NA	NA
NA	NA	NA	NA
NA	NA	NA	NA
NA	NA	NA	NA
NA	NA	NA	NA
NA	NA	NA	NA
NA	NA	NA	NA
NA	NA	NA	NA
NA	NA	NA	NA
NA	NA	NA	NA
NA	NA	NA	NA
NA	NA	NA	NA
NA	NA	NA	NA
NA	NA	NA	NA
NA	NA	NA	NA
NA	NA	NA	NA
365.36	up	5.61	2.07E-09
363.06	up	4.19	2.45E-10
144.84	up	4.11	1.77E-05
173.12	up	3.83	1.74E-06
309.50	up	3.63	2.09E-07
184.29	down	3.56	2.40E-14
114.82	down	3.23	1.98E-10
159.53	up	3.13	4.12E-07
301.23	up	3.07	1.83E-10

335.29	up	2.87	1.07E-05
95.95	down	2.86	9.41E-06
403.96	up	2.77	3.03E-08
355.36	up	2.53	2.69E-07
212.61	up	2.47	1.67E-04
252.65	up	2.35	2.14E-06
68.98	up	2.26	5.96E-03
201.83	up	2.16	2.54E-03
201.83	up	2.16	2.54E-03
187.16	up	2.07	2.02E-06
413.58	up	2.04	8.58E-09
178.52	up	1.96	6.79E-02
76.49	up	1.93	2.65E-01
174.86	up	1.73	1.64E-04
104.40	up	1.72	7.08E-03
298.35	down	1.70	3.54E-04
242.35	down	1.68	3.67E-04
372.36	up	1.66	1.88E-04
490.92	up	1.59	3.15E-06
112.92	down	1.47	7.06E-03
342.14	up	1.47	2.44E-04
34.66	up	1.37	1.01E-02
90.11	up	1.33	4.95E-03
388.24	up	1.32	1.70E-03
235.55	up	1.31	5.02E-02
389.64	down	1.24	1.25E-02
176.57	down	1.21	3.15E-02
146.41	up	1.18	3.90E-01
146.41	up	1.18	3.90E-01
188.43	up	1.11	1.59E-01
145.90	up	1.08	2.39E-01
NA	NA	NA	NA
NA	NA	NA	NA
NA	NA	NA	NA
594.19	up	1.46	3.81E-06
698.13	up	1.44	4.34E-04
1437.31	up	1.21	2.53E-03
2254.91	up	1.20	6.14E-07
4970.87	down	1.19	1.60E-06
918.37	up	1.16	4.68E-03
1337.52	down	1.15	2.01E-02
1139.58	up	1.14	7.43E-03
2844.16	down	1.13	1.91E-02
2022.54	up	1.12	1.88E-02
1237.69	down	1.12	2.00E-02
986.24	down	1.11	5.36E-02
920.31	down	1.08	3.57E-01
652.50	up	1.07	4.41E-02
559.99	up	1.07	3.30E-01
4137.91	down	1.06	7.16E-02
4137.91	down	1.06	7.16E-02
4137.91	down	1.06	7.16E-02
4137.91	down	1.06	7.16E-02
709.66	up	1.03	5.81E-01
3555.49	up	1.01	2.59E-02
222.76	up	3.05	5.66E-05

20.11	up	1.55	3.24E-03
115.68	down	1.46	2.24E-03
192.89	up	1.44	1.10E-03
255.19	down	1.29	9.38E-03
421.76	down	1.27	7.08E-02
357.95	up	1.01	1.79E-01
